# Expression of sugarcane COBRA-Like genes, *ScBC1* and *ScBC1L2*, increases plant biomass

**DOI:** 10.1186/s12870-025-07910-y

**Published:** 2025-12-15

**Authors:** Samara Cardoso, Júlia Dias Maloste, Leandro Lira Souza, Talia Jacobson, Renato Vicentini, André Ferraz, Cătălin Voiniciuc, Elisson Romanel, Steven A. Whitham, Tatiane da Franca Silva

**Affiliations:** 1 Plant Genomics and Bioenergy Laboratory (PGEMBL), Department of Biotechnology, Lorena School of Engineering, University of São Paulo (EEL-USP), Lorena, SP 12602-810 Brazil; 2 Wood Science Laboratory, Department of Biotechnology, Lorena School of Engineering, University of São Paulo (EEL-USP), Lorena, SP 12602-810 Brazil; 3 Horticultural Sciences Department, University of Florida, Gainesville, FL 32611 USA; 4 Department of Plant Pathology, Entomology and Microbiology, Iowa State University, Ames, IA 50011 USA; 5 Systems Biology Laboratory, Institute of Biology, University of Campinas, São Paulo, 13083- 872 Brazil

**Keywords:** Cellulose, Biomass, COBRA (genes/proteins), *Saccharum* (sugarcane), Cell wall

## Abstract

**Background:**

Cellulose is a major determinant of plant biomass yield and quality, with significant industrial relevance. COBRA proteins are established regulators of cellulose deposition and cell wall organization; however, their roles in sugarcane (Saccharum spp.) growth and development remain uncharacterized.

**Results:**

A genome-wide analysis identified 50 sugarcane *COBRA* genes grouped into 11 unigenes, with *ScBC1* (*Brittle Culm 1*) and *ScBC1L2* (*Brittle Culm Like 2*) highly expressed in stems. Phylogenetic analysis placed *ScBC1* with secondary cell wall-associated genes, including maize *ZmBK2*, while *ScBC1L2* clustered with primary cell wall-related genes. Transient expression of *ScBC1* and *ScBC1L2* in *Nicotiana benthamiana* increased leaf biomass and epidermal cell size, with *ScBC1* having the strongest effect. Virus-induced gene silencing of *ZmBK2* in maize reduced biomass, cellulose content, and cell area, and altered the expression of *Cellulose Synthase* (CesA) genes. Genes related to cell wall remodeling, including *Expansin*, *β-Galactosidase*, and *Polygalacturonase*, were differentially expressed in *ZmBK2*-silenced leaves, suggesting compensatory responses.

**Conclusions:**

These findings indicate a conserved role of the *ScBC1–ZmBK2* subclade in cellulose deposition and cell expansion, identifying *ScBC1* as a promising target for improving sugarcane biomass.

**Supplementary Information:**

The online version contains supplementary material available at 10.1186/s12870-025-07910-y.

## Background

Cellulose is a structural polysaccharide in plants that is a key raw material for a wide range of industries. Its versatility allows for extensive applications, including the production of paper, packaging, and textiles, as well as its use in disposable hygiene products, food, pharmaceuticals, adhesives, biofuels, and construction materials [1–3]. In plants, cellulose plays a crucial role in defining the physical and chemical properties of cell walls. Its highly ordered linear arrangement in microfibrils results in significant crystallinity, conferring mechanical strength, rigidity, and structural integrity to the cell wall. This organization supports plant architecture, enabling upright growth and resistance to external forces. Besides its structural function, cellulose also acts as a protective barrier against pathogens and environmental stressors, enhancing plant resilience [4–7].

A variety of plant proteins are involved in biological processes associated with the synthesis and organization of cellulose microfibrils. Among them, members of the COBRA family play a significant role in the formation of primary and secondary cell walls. Initially identified in *Arabidopsis thaliana*, COBRA proteins essentially influence the patterning of cellulose microfibril arrangement [8–10].

The *Arabidopsis COBRA* gene family consists of 12 members, including *COBRA* (*COB)* and *COBRA-like* (*COBL1* to *COBL11*), which can be classified into two distinct subgroups based on total protein length, sequence identity, and structural domain organization. The first subgroup, comprising *AtCOBL1* to *AtCOBL6*, encodes proteins with high structural similarity to the original COBRA protein. In contrast, the second subgroup, composed of *COBL7* to *COBL11*, exhibits an additional N-terminal domain, resulting in proteins approximately 45% longer than the other members of the family [11].

In general, COBRA family proteins share three conserved domains: (i) a carbohydrate-binding module (CBM) located in the N-terminal region; (ii) a cysteine-rich CCVS domain, potentially involved in disulfide bond formation; and (iii) a hydrophobic C-terminal region that mediates plasma membrane anchoring via a glycosylphosphatidylinositol (GPI) anchor [8, 9, 11]. The CBM domain present in COBRA mediates interactions with cellulose polymers synthesized by the multimeric rosette complexes of cellulose synthase (CesA) proteins. These complexes, embedded in the plasma membrane, catalyze the polymerization of (1→4)-β-D-glucan chains, which are extruded into the cell wall and assembled into well-organized cellulose microfibrils [9, 11–13].

In grasses, members of the COBRA gene family have been named based on the phenotypic characteristics observed in mutants, such as *Brittle Stalk-like 2* (*ZmBK2*) in maize and *Brittle Culm 1* (*OsBC1*) in rice [14, 15]. Silencing *COBRA* genes in rice, maize, and *Arabidopsis* plants has been shown to reduce mechanical strength and cellulose deposition [9, 15–18]. Additionally, *COBRA* gene family members are key regulators of various biological processes, including the anisotropic expansion during the leaf morphogenesis [19, 20], formation of the cell wall in epidermal and collenchyma cells [21], and the regulation of root development and stress response [22]. Although the importance of COBRA protein in cellulose biosynthesis and deposition in the plant cell wall is recognized, relatively little is known about the COBRA gene family in economically relevant biomass plant species, such as sugarcane.

Sugarcane is an important crop around the globe, particularly in South America and Asia. Brazil is the world’s largest producer of sugarcane [23, 24], which plays key role in the national economy. It belongs to the genus *Saccharum* and the family *Poaceae*, which includes other economically relevant grasses such as maize (*Zea mays*) and sorghum (*Sorghum bicolor*). Sugarcane features succulent stems or stalks, from which a sucrose-rich juice can be extracted. While the juice is used to produce a variety of industrial products, including sugar and bioethanol [25], the stalk biomass is an important raw material for cellulose-based products [26, 27]. Investigating the COBRA gene family in sugarcane is expected to reveal valuable biotechnological applications, offering insights into the development of this biomass with tailored cellulose content and quality. Such advancements could drive innovation in bio-based industries, including biofuels, paper production, and sustainable materials.

In this study, we conducted a genome-wide characterization of the *COBRA* gene family in sugarcane and identified two key genes, *ScBC1* and *ScBC1L2*, involved in modulating cell wall architecture. Using the foxtail mosaic virus (FoMV) vector system, we performed transient expression in *N. benthamiana* and virus-induced gene silencing (VIGS) in maize, targeting *ZmBK2* and *ZmBK2L3*, orthologs of *ScBC1* and *ScBC1L2*, respectively. Transient expression assays showed that both genes, particularly *ScBC1*, influence leaf cell size and biomass accumulation. VIGS of *ZmBK2* further confirmed its conserved role in regulating cell size and cell wall dynamics. Transcript analysis in silenced plants revealed altered expressions of genes related to cell wall remodeling, suggesting compensatory responses to reduced cellulose. These results position *ScBC1* as a promising candidate for improving sugarcane biomass and provide the first evidence linking *ZmBK2* to cell size and cell wall loosening pathways.

## Methods

### Identification, annotation, and phylogenetic analysis

The COBL domain (PF04833), retrieved from Pfam (http://pfam.xfam.org/), was used as a query in the Phytozome13 database (https://phytozome-next.jgi.doe.gov/). Putative COBRA-like genes were identified using the integrated HMMER tool, applying the gathering threshold and requiring full domain coverage. Candidate sequences were retrieved for *A. thaliana* TAIR10, *S. bicolor* V3.1.1, *Z. mays* V4, *O. sativa* V7.0, and Saccharum officinarum × S. spontaneum R570 v2.1 [28]. Multiple sequence alignment was performed using the MUSCLE algorithm [29] in MEGA11 [30] software, followed by phylogenetic tree using the Maximum Likelihood method, with a bootstrap value set to 100 [31]. Putative orthologous genes across plant species were identified based on their position within the same clade in the phylogenetic analysis. The identifiers of all genes used in this work are provided in Table S1.

### Transcriptomic data analysis

The in silico analysis of COBRA gene expression in sugarcane was conducted by quantifying transcript abundance in RNA sequencing libraries from internodes of two sugarcane lines IACSP04-065 and IACSP04-627 [32], and the SUCEST (Sugarcane EST project) transcriptome database [33, 34], which comprises transcripts from various plant organs. The SUCEST dataset [33, 34] and RNA-seq dataset [35] were normalized using the number of reads per sequence relative to the whole and transcripts per million (TPM), respectively. Expression values were further transformed to a log2 scale. Heatmaps were generated using RStudio (https://www.rstudio.com/) with hierarchical clustering based on Euclidean distance and the complete linkage method.

### Quantitative PCR expression profile analysis in sugarcane hybrids

Of the eleven experimental sugarcane hybrids characterized by Masarin et al. (2011), cultivated for 12 months at the Lorena School of Engineering (EEL/USP), hybrids 89 (H89) and 140 (H140) were selected for the present study. These hybrids were chosen due to their contrasting chemical composition and bagasse digestibility [36–38]. The sugarcane material was collected and processed as described [39]. The fifth internode of 1-year-old plants, as well as leaves and stems from young plants (3 months post-planting), were harvested from both sugarcane hybrids. The collected samples were immediately frozen in liquid nitrogen to preserve material integrity. Total RNA was extracted from 300 mg of each macerated tissue using the Concert™ Plant RNA Reagent (Invitrogen). The purity and concentration of the extracted RNA were assessed using a spectrophotometer, and RNA integrity was verified through agarose gel electrophoresis. To eliminate potential genomic DNA contamination, the RNA samples were treated with RQ1 RNase-free DNase (Promega). First-strand cDNA synthesis was performed using a 100 µM oligo(dT)24 primer and the SuperScript™ III First-Strand Synthesis System (ThermoFisher).

For RT-qPCR analysis, specific primers for two putative sugarcane COBRA genes *ScBC1* and *ScBC1L2* were designed within the last exon and 3’ UTR region (Table S2) using the OligoAnalyzer™ tool Integrated DNA Technologies (https://www.idtdna.com/). Quantitative real-time PCR reactions were conducted in a 7500 Fast Real-Time PCR System (Life Technologies, Thermo Fischer) employing SYBR Green. Each reaction consisted of 2.5 µL of cDNA (diluted 25 times), 5 µL of Maxima SYBR Green/Rox qPCR Master Mix (Life Technologies, Thermo Fischer), 0.6 µM of primers, and a final reaction volume of 20 µL. The reaction conditions were set to 50 °C for 2 min, 95 °C for 10 min, followed by 40 cycles of 95 °C for 15 s and 60 °C for 1 min. For data normalization, the reference genes *glyceraldehyde-3-phosphate dehydrogenase* (*GAPDH*) and *polyubiquitin* (*PUB*) [40, 41] were used in the analysis of sugarcane *COBL* genes. Relative expression was calculated using the ∆CT method [42]. Two biological replicates were analyzed, each with three technical replicates per cDNA sample.

### Determination of the crystallinity index of sugarcane hybrids rind

To determine the crystallinity of rind from internode 5, tissue was collected as described [39], samples from H89 and H149 were macerated in liquid nitrogen, stored frozen at −80 °C, and then dried in an oven. The crystallinity index (CI) was measured as previously described [43], utilizing the peak width deconvolution method (DPHM). X-ray diffraction data were collected using a diffractometer (XRD–6000, Shimadzu) operating at room temperature with CuKα radiation and a graphite monochromator. The peaks obtained from the diffraction spectrum were analyzed by deconvolution using the Gaussian function of Origin software (OriginLab, version 2017). Analyses were performed in technical triplicates and biological duplicates.

### Construction of FoMV-based vectors for *ScBC1* and ScBC1L2

To enable heterologous transient expression of *ScBC1* and *ScBC1L2*, full-length cDNAs were cloned into the pFoMV-DC vector [44] at the *HpaI* restriction site, generating FoMV-VOX (virus-induced overexpression) vectors. The *ScBC1* cDNA clone (SCCCST3002E04.g), used for FoMV-VOX construction, was obtained from the SUCEST library at the Center for Biological Resources and Genomic Biology (CREBIO), UNESP. The coding region of *ScBC1L2* was amplified from sugarcane RNA using reverse transcription polymerase chain reaction (RT-PCR) as described above. The full-length coding sequences of *ScBC1* (1380 bp) and *ScBC1L2* (1173 bp) were amplified using gene-specific primers (Table S3). To incorporate a V5 tag at the protein C-terminal region, the purified PCR products underwent a second PCR cycle, utilizing the primers ScBC1-VOX-F/ScBC1-VOX-R for *ScBC1*, and ScBC1L2-VOX-F/ScBC1L2-VOX-R for *ScBC1L2* (Table S3).

The NEBuilder HiFi DNA Assembly Cloning Kit (New England Biolabs) was used to insert the *ScBC1* and *ScBC1L2* coding sequences fused with the V5 tag into pFoMV-DC. For this, a third round of PCR was performed using the primers ScBC1L2-VOX-F/V5-FoMV-R for *ScBC1L2* and ScBC1-VOX-F/V5-FoMV-R for *ScBC1* (Table S3), amplifying the inserts flanked by homologous sequences to the virus vector. The final product for both genes was inserted into pFoMV-DC that was linearized with *HpaI*, following the manufacturer’s instructions.

For the virus-induced gene silencing (VIGS) assay, specific antisense fragments (~ 300 bp) of the *ScBC1* and *ScBC1L2* genes, flanked by sequences homologous to pFoMV-DC, were synthesized (Integrated DNA Technologies-IDT). The pFoMV vector was linearized using *MluI*, and the synthesized fragments were inserted using the NEBuilder HiFi DNA Assembly Cloning Kit (New England Biolabs). All PCR reactions described in this section were performed using Phusion^®^ High-Fidelity DNA Polymerase (New England Biolabs). The constructs FoMV-VOX-ScBC1, FoMV-VOX-ScBC1L2, FoMV-VIGS-ScBC1, and FoMV-VIGS-ScBC1L2 (Figure S1) were confirmed by DNA sequencing and individually introduced into *Agrobacterium tumefaciens* strain GV3101.

### Agroinfiltration of *N. benthamiana*

FoMV clones were inoculated by direct infiltration of transformed *Agrobacterium tumefaciens* strain GV3101 cultures into *N. benthamiana* leaves as described [45]. Bacterial cultures were grown in Luria-Bertani medium supplemented with 20 µM acetosyringone until optical densities (OD600): 1.0 for *FoMV-VOX-ScBC1*, 1.2 for *FoMV-VOX-ScBC1L2*, 1.1 for *pFoMV-DC*, 1.0 for *FoMV-DC-GFP*, 1.0 *FoMV-VIGS-ScBC1*, and *1.0 FoMV-VIGS-ScBC1L2*. Bacterial cells were harvested by centrifugation at 8,000 × g for 10 min and resuspended in an infiltration buffer composed of 10 mM MES, 10 mM MgCl2, and 100 µM acetosyringone (pH 5.6), and incubated at 20 °C for one hour. The *A. tumefaciens* suspension for each construct was infiltrated into the leaves of *N. benthamiana* plants that were grown in a growth chamber at 22 °C for six weeks under a 16 h of light/8 h dark photoperiod. Following agroinfiltration, the plants were kept in the growth chamber for five days. Total RNA was extracted from *N. benthamiana* infiltrated leaves, and reverse transcription reactions were performed as described above. The resulting cDNA was used as a template for PCR amplification using Phusion^®^ High-Fidelity DNA Polymerase (New England Biolabs). Expression of the virus vectors was analyzed using the primers FoMV-MCS-VOX-F and FoMV-MCS-VOX-R for FoMV-VOX-ScBC1 and FoMV-VOX-ScBC1 and FoMV-MCSI-VIGS-F and FoMV-MCSI-VIGS-R for FoMV-VIGs-ScBC1 and FoMV-VIGs-ScBC1L2 (Table S3). The *Actin* gene (*NbACT09*) [46] was used as an internal control. PCR products were resolved by electrophoresis on a 0.8% agarose gel.

### Protein extraction and Western blot assays

Total protein was extracted from *N. benthamiana* leaf samples of agroinfiltrated plants with *pFoMV-DC*, *FoMV-DC-GFP*, *FoMV-VOX-ScBC1*, and *FoMV-VOX-ScBC1L2*. Approximately 0.35 g of leaf tissue was collected with a corer and then macerated using a tissue grinder with pestle. The macerated tissue was homogenized in 700 µl of extraction buffer containing 50 mM NaCl, 20 mM Tris (pH 7.5), 1 mM EDTA, 0.1% Triton X-100, 10% glycerol, 5 mM DTT, 2 mM NaF, 1 mM PMSF, and a protease inhibitor cocktail (1x). Following centrifugation, the supernatant was mixed with SDS-PAGE loading buffer and heated at 95 °C for 5 min. Twenty µl of each boiled sample was loaded onto an SDS-PAGE gel, followed by protein transfer to a membrane for Western blotting. For detection, a V5 Tag Monoclonal Antibody (2F11F7) (Invitrogen) was used at a 1:5000 dilution, followed by a secondary HRP-conjugated Affinipure Goat Anti-Mouse IgG (H + L) antibody at the same dilution, with overnight incubation. For GFP detection, the GFP Antibody (MA5-15256) was applied at a 1:3000 dilution.

### Inoculation of maize with FoMV-VIGS and VOX constructs

Leaves of *N. benthamiana* agroinfiltrated with the viral vectors *FoMV-VOX* (*FoMV-VOX-ScBC1* and *FoMV-VOX-ScBC1L2*) and *FoMV-VIGS* (*FoMV-VIGS-ScBC1* and *FoMV-VIGS-ScBC1L2*), and the empty vector control (*pFoMV-DC*) were used as inoculum for maize seedlings at seven days post-germination, when the plants had developed a single true leaf. In addition, Mock-inoculated plants (with buffer solution only) were included as a second negative control. The maize leaves were dusted with 600-mesh carborundum to create microabrasions, facilitating viral infection. The agroinfiltrated *N. benthamiana* leaves were then ground in 50 mM potassium phosphate buffer (KH₂PO₄), pH 7.0, using a mortar and pestle at a 1:10 (weight/volume) ratio. The resulting liquid inoculum was rub-inoculated onto the leaves of each maize seedling using the pestle. After the inoculum dried on the leaves, they were rinsed by spraying with water. At 14 days post inoculation (dpi), the plants were phenotypically analyzed, and leaf samples were collected for RNA extraction to confirm the expression of the viral constructs in maize. Expression of the virus vectors was analyzed using the primers FoMV-MCS-VOX-F and FoMV-MCS-VOX-R for FoMV-VOX-ScBC1 and FoMV-VOX-ScBC1L2, and FoMV-MCSI-VIGS-F and FoMV-MCSI-VIGS-R for FoMV-VIGs-ScBC1 and FoMV-VIGs-ScBC1L2 (Table S3). The *Actin* gene (*ZmACT1*), using primers listed in Table S2, was employed as an internal control. PCR products were resolved by electrophoresis on a 0.8% agarose gel.

Silencing of the *ZmBK2* and *ZmBK2L3* genes in maize plants inoculated with FoMV-VIGS constructs (FoMV-VIGS-ScBC1 and FoMV-VIGS-ScBC1L2) was evaluated by RT-qPCR, as described above. Transcript quantification was performed using gene-specific primers listed in Table S2. Expression levels of *ZmBK2* and *ZmBK2L3* were normalized to the reference gene *Zmβ-tubulin* (Lin et al., 2014), and relative expression was calculated using the 2^−ΔΔCT^ method [47, 48], with the Mock 2 sample used as the calibrator.

### Physiological evaluation of *N. benthamiana* expressing FoMV-VOX constructs and maize plants silenced via FoMV-VIGS

The dry mass of 110 leaves from 22 plants of *N. benthamiana* agroinfiltrated with FoMV-VOX constructs (FoMV-VOX-ScBC1, FoMV-VOX-ScBC1L2, FoMV-DC-GFP, and empty vector) was determined by drying the samples in an oven at 35 °C until a constant weight. At 14 dpi, the aerial shoots of the maize plants (14 dpi) silenced for *ZmBK2* and *ZmBK2L3* using FoMV-VIGS constructs (FoMV-VIGS-ScBC1 and FoMV-VIGS-ScBC1L2) were harvested and oven-dried at 60 °C until constant weight was achieved. The final dry mass was expressed in grams per plant. In addition, silenced maize plants were also analyzed for plant height and leaf area as described [49].

### Microscopy and cellular area quantification

*N. benthamiana* leaves agroinfiltrated with FoMV-VOX-ScBC1, FoMV-VOX-ScBC1L2, and FoMV-DC-GFP constructs and expressing the inserts were numbered sequentially from the top to the base of the plant. For microscopic analysis of cellular area, the seventh leaves positioned at the same developmental stage were collected from 22 agroinfiltrated plants for each construct. The total area of the abaxial epidermal cells was determined as previously described [50] with some modifications. A 1.0 cm^²^ square was excised from the agroinfiltrated region of each leaf and analyzed using an Olympus BX53 microscope under brightfield field mode. Ten cells from each leaf were counted, resulting in a total of 220 cells per construct. For image processing and cellular area measurement, CellSens 8.0 software was employed. GFP visualization was performed using the fluorescence mode, an X-CITE 120 LED light source, and EYFP filter.

**Fig. 1:**
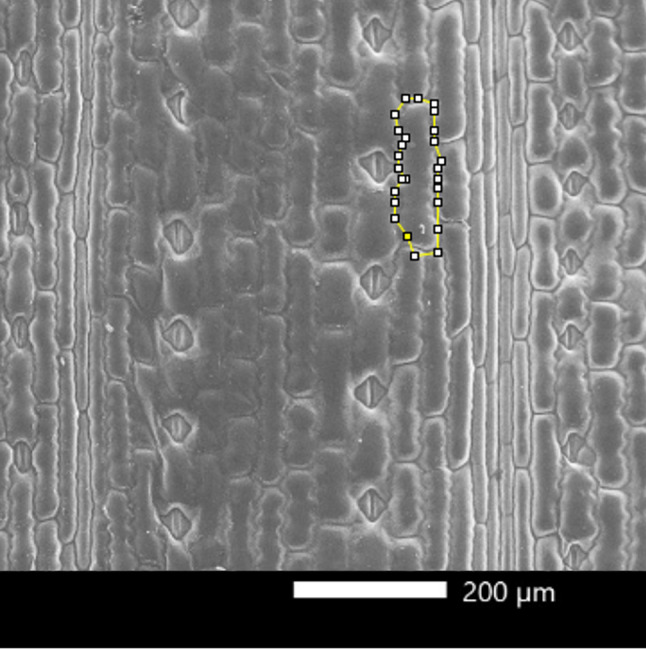
Representative scanning electron microscopy (SEM) image of the adaxial epidermis of maize leaves. The area of a bulliform cell is highlighted, which was quantified using ImageJ software. Images captured at 65× magnification; scale bar = 200 μm

Maize plants at 14 dpi with pFoMV, FoMV-VIGS-ScBC1 and FoMV-VIGs-ScBC1L2, showed *ZmBk2* and *ZmBk2L3* silencing, respectively, as well as Mock controls had their fourth leaf (counting from base to apex) collected for epidermal area analysis by scanning electron microscopy (SEM). For SEM analysis, 2 cm² leaf samples were excised 8 cm from the base of the right side of the leaf. The prepared samples were observed using a SEM, model JEOL NeoScope JCM-7000. The analysis was conducted at 65× magnification, enabling detailed visualization of cell morphology on the adaxial surface of the leaves. Pavement cell measurements were performed using ImageJ software in the bulliform cell (Fig. 1), individual cell boundaries were manually outlined, and the areas were calculated in square micrometers (µm²). For each sample, measurements were taken from three distinct regions to ensure data reproducibility and accuracy. A total of 20 cells per plant were counted.

### RT-qPCR analysis of cell wall-related gene expression in maize plants silenced via FoMV-VIGS

Transcript levels of cell wall-related genes, including the secondary cell wall cellulose synthase *ZmCesA10*, *ZmCesA11*, and *ZmCesA12* [51] were evaluated in both *ZmBK2* and *ZmBK2L3*-silenced maize plants. Additionally, the expression of *ZmBGAL1* (β-galactosidase) [52], *ZmPG44* (polygalacturonase) [53], and *ZmEXP10* (expansin) [54] were specifically analyzed in *ZmBK2*-silenced plants. All transcript analyses were performed by RT-qPCR, as described above. Gene expression was normalized to the reference gene *Zmβ-TUB* [55], and relative transcript levels were calculated using the 2^–ΔΔCT^ method [47, 48], with the Mock 2 sample serving as the calibrator. Specific primers employed in RT-qPCR are listed in Table S2.

### Alcohol-insoluble residue (AIR) Preparation and starch removal

Leaves of agroinfiltrated *N. benthamiana* expressing *ScBC1* and *ScBCL2* and *ZmBK2*-silenced maize plants were ground to a fine powder using a mortar and pestle under liquid nitrogen. Afterward, insoluble residues were isolated through three sequential washes with 1mL of 70% ethanol (v/v) followed by centrifugation at 20,000 *g* for 3 min. After washing the AIR with 1:1 (v/v) chloroform methanol, followed by 1 mL of acetone, samples were dried under a stream of air at 45 °C for 1 h. To destarch each sample, 2 to 3 mg of AIR was resuspended in 0.2 M phosphate buffer (pH 7.0) supplemented with 1 µL of α-Amylase (Sigma Aldrich Cat# A3403), 10 µL of Pullulanase (Sigma Aldrich Cat# E2412), and sodium azide (as an antimicrobial agent). AIR was incubated at 45 °C at 300 rpm in neoLab thermomixer for 30 min. Subsequently, samples were cooled on ice and mixed overnight (20 h, 250 rpm and 37 °C) in an Eppendorf 42 shaking incubator. The next day, the destarched AIR pellet obtained after 2 min at 20,000 *g* was washed once with 1.5 mL of water, followed by 1.5 mL of acetone. The samples were concentrated under pressurized air at 45 °C for 1 h.

### Crystalline and amorphous polymer hydrolysis and detection

A two-step hydrolysis of total and matrix-only polysaccharides was performed using 1 mg aliquots of destarched AIR [56, 57], alongside monosaccharide standards. For Saeman hydrolysis samples, 50 µL of 72% (w/w) sulfuric acid was added and incubated at room temperature for 1 h. After incubation, 450 µL of 30 µg/mL Ribose internal standard, and 970 µL of water were added. To the matrix samples and standards, 400 µL of water, 450 µL of the internal standard, and 50 µL of 72% (w/w) were added. All samples and standards were homogenized in a Retsch mill for 2:30 min at 30 Hz before incubating for 60 min at 120 °C. After hydrolysis, monosaccharides were quantified using high-performance anion-exchange chromatography (HPAEC-PAD) as specified by [57, 58], using a Metrohm ion chromatography gradient and a specified gradient.

### Statistical data analysis

The statistical analysis of quantitative real-time PCR, dry leaf mass production, cell area quantification assays, leaf area and chemical characterization, and plant height was conducted using one-way ANOVA followed by Tukey’s test. Differences were considered statistically significant at *p* < 0.05. All analyses were performed using Statistica version 14.0.015 (TIBCO Software Inc., USA).

## Results

### Identification and phylogenetic analysis of the COBRA gene family in sugarcane

To identify *COBRA* genes in the *Saccharum* spp. genome (Phytozome, *Saccharum officinarum × spontaneum* R570 v2.1), a genome-wide survey was conducted using the conserved COBL domain (Pfam04833). Following domain-based screening, 50 out of 63 candidate sugarcane sequences were confirmed to contain the characteristic COBL domain (Table S1). The number of putative COBRA protein-coding genes was considerably higher than the 11 genes reported in Arabidopsis, 11 reported in rice, 10 in maize, and 10 in sorghum [11, 14, 59, 60].

**Fig. 2:**
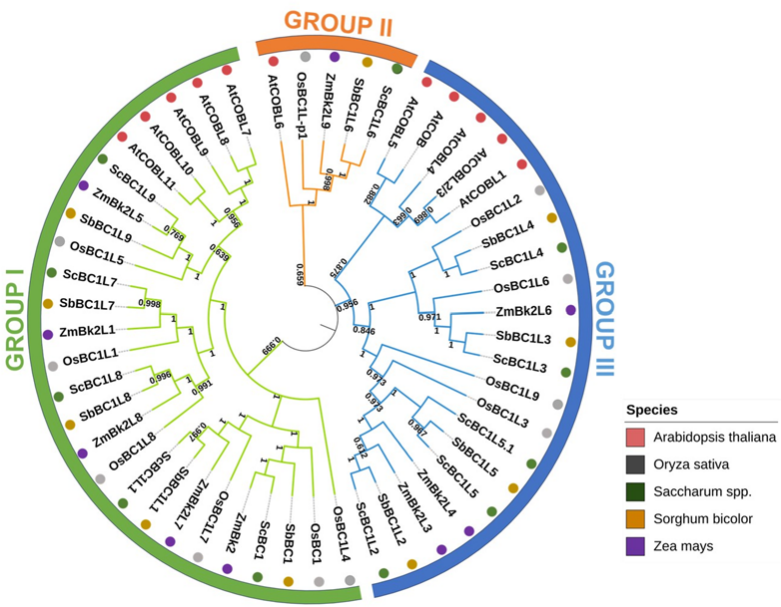
Phylogenetic analysis of COBRA protein sequences from *Saccharum spp. *Phylogenetic relationships based on amino acid sequences of COBRA family proteins from *Saccharum* spp. (green), *Sorghum bicolor* (yellow), *Zea mays* (purple), *Oryza sativa* (gray), and *Arabidopsis thaliana* (red). The phylogenetic analysis was performed using the Maximum Likelihood method. Bootstrap values from 1000 replicates were used to assess the robustness of the tree

To evaluate the phylogenetic relationships among *COBRA* genes in sugarcane, *Arabidopsis*, rice, maize, and sorghum, a phylogenetic analysis was performed using the Maximum Likelihood method (Fig. 2). Redundant sugarcane sequences clustering within the same clade were omitted from the phylogenetic analysis. Instead, a single representative sequence (unigene) from each group, named based on orthologous to sorghum, was used (Table S1). The results revealed a clear division of the COBRA family into three well-supported major clades, designated as Groups I, II, and III, consistent with previous reports (Fig. 2) [59, 61].

These major clades include both *Arabidopsis* and grass genes, subdivided into distinct subclades, reflecting the evolutionary relationship between eudicots and grasses. Groups I and III presented grass-exclusive subclades, suggesting divergence from ancestral lineages distinct from those of *Arabidopsis* (Fig. 2). One of the grass-specific subclades in Group I includes important previously characterized *COBRA* genes in grasses, such as *ZmBK2* (*Brittle Stalk-like 2*) in maize, *OsBC1* (*Brittle Culm 1*) in rice, and *SbBC1* in sorghum [14, 60, 62]. Genome distribution analysis of the 11 sugarcane COBRA unigenes revealed that they are predominantly located on Groups 1 and 5 polyploid chromosomes (1 A, 1B, 1E, 1 F, 5 A and 5 F), with no evidence of tandem duplication (Table S1 and Figure S2).

### Expression analysis of COBRA genes in sugarcane

To identify functional *COBRA* genes in sugarcane, we first analyzed transcript expression in the SUCEST database [33, 34] and the RNA-seq data from stem tissue of two sugarcane lines IACSP04-065 and IACSP04-627 [35]. Correspondence between *COBRA* gene sequences of *Saccharum* spp. identified in the PHYTOZOME v13.0 database, and their homologous sequences in the SUCEST and RNA-seq databases are listed in Table S4. The nomenclature of the sugarcane genes was established based on orthology to the *Sorghum bicolor* COBRA genes.

**Fig. 3:**
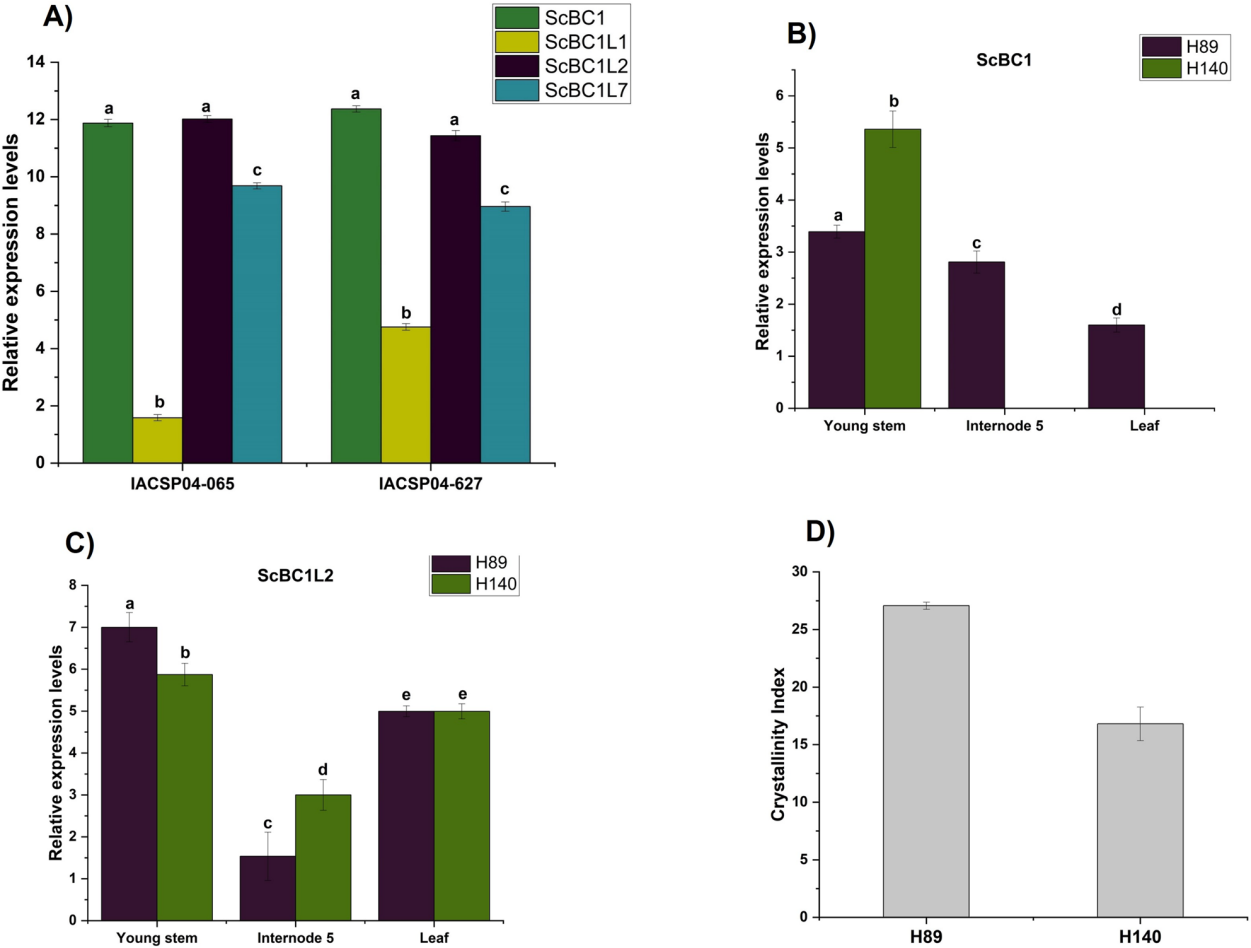
Expression analysis of sugarcane COBRA genes and cellulose crystallinity index of H89 and H140hybrids. **A** Quantification of COBRA gene transcript expression from RNA-seq datasets of sugarcane correlates with contrasting lignin contents (IACSP04-065 and IACSP04-627) (Vicentini et al., 2015). **B** and **C **Expression profiles of *ScBC1* and *ScBC1L2* genes in H89 and H140 hybrids, respectively. Relative expression levels of *ScBC1* and *ScBC1L2* were determined by RT-qPCR in leaves and young stems from 3-month-old plants, internodes from 1-year-old first ratoon plants. The relative expression was calculated using the ∆CT method (CtCOBRA − CtGAPDH/PUB). **D** Cellulose crystallinity index (CI) determined for cortex samples from the fifth internode of the H89 and H140 hybrids. Statistical differences between samples were determined by one-way ANOVA followed by Tukey’s test (*p* < 0.05). Bars represent standard errors (SD)

Five putative *COBRA* genes, *ScBC1*, *ScBC1L7*, *ScBC1L1*, *ScBC1L3*, and *ScBC1L5.1* were identified in the SUCEST database (Figure S3). However, only four genes were detected in RNA seq data (*ScBC1*, *ScBC1L7*, *ScBC1L2*, and *ScBC1L1*) (Fig. 3A). Most of the identified sequences were expressed in both databases, except for *ScBC1L5.1* and *ScBC1L2*. *ScBC1L5.1* exhibited low expression levels exclusively in the SUCEST libraries (Figure S3), while *ScBC1L2* was detected only in the RNA-seq dataset (Fig. 3a and Figure S3). Among the analyzed genes in the RNA-seq data, *ScBC1* and *ScBC1L2* exhibited the highest expression levels in the internodes of both sugarcane lines (Fig. 3A). In agreement, *ScBC1* showed the highest expression in the stem bark and the fourth internode of adult plants (SUCEST database) (Figure S3).

The in silico expression analysis enabled the identification of expressed *COBRA* genes within the highly redundant sugarcane gene family, particularly those active in the stem and internodes (*ScBC1* and *ScBC1L2*), the primary organ of industrial importance in this crop. Due to their elevated expression levels in the in silico analysis (Fig. 3A and S3), *ScBC1* and *ScBC1L2* were selected for expression profiling using RT-qPCR assay in different organs (fifth internode of 1-year-old plants, young stems, and leaves from 3-month-old plants) (Fig. 3B and C). The transcript abundance analyses were conducted in two non-commercial sugarcane hybrids, H89 and H140, which present contrasting chemical compositions [36–38]. These traits influence lignocellulosic biomass recalcitrance, which can also be associated with multiple factors such as cell type, maturation, and cellulose crystallinity index [37, 38]. Transcripts of *ScBC1* and *ScBC1L2* showed significantly higher expression (*p* < 0.05) in the young stem, a rapidly growing tissue, in both sugarcane hybrids (Fig. 3B and C), suggesting a potential role for both genes in cell wall architecture during stem elongation. Interestingly, in this tissue, *ScBC1* expression was higher in H140, whereas *ScBC1L2* was higher in H89. In the current study, the cellulose crystallinity index (CI) was determined for rind samples from the fifth internode of both hybrids (Fig. 3D), revealing that H89 exhibited a higher CI than H140.

### Transient Expression of *ScBC1* and ScBC1L2 in *N. benthamiana*

**Fig. 4:**
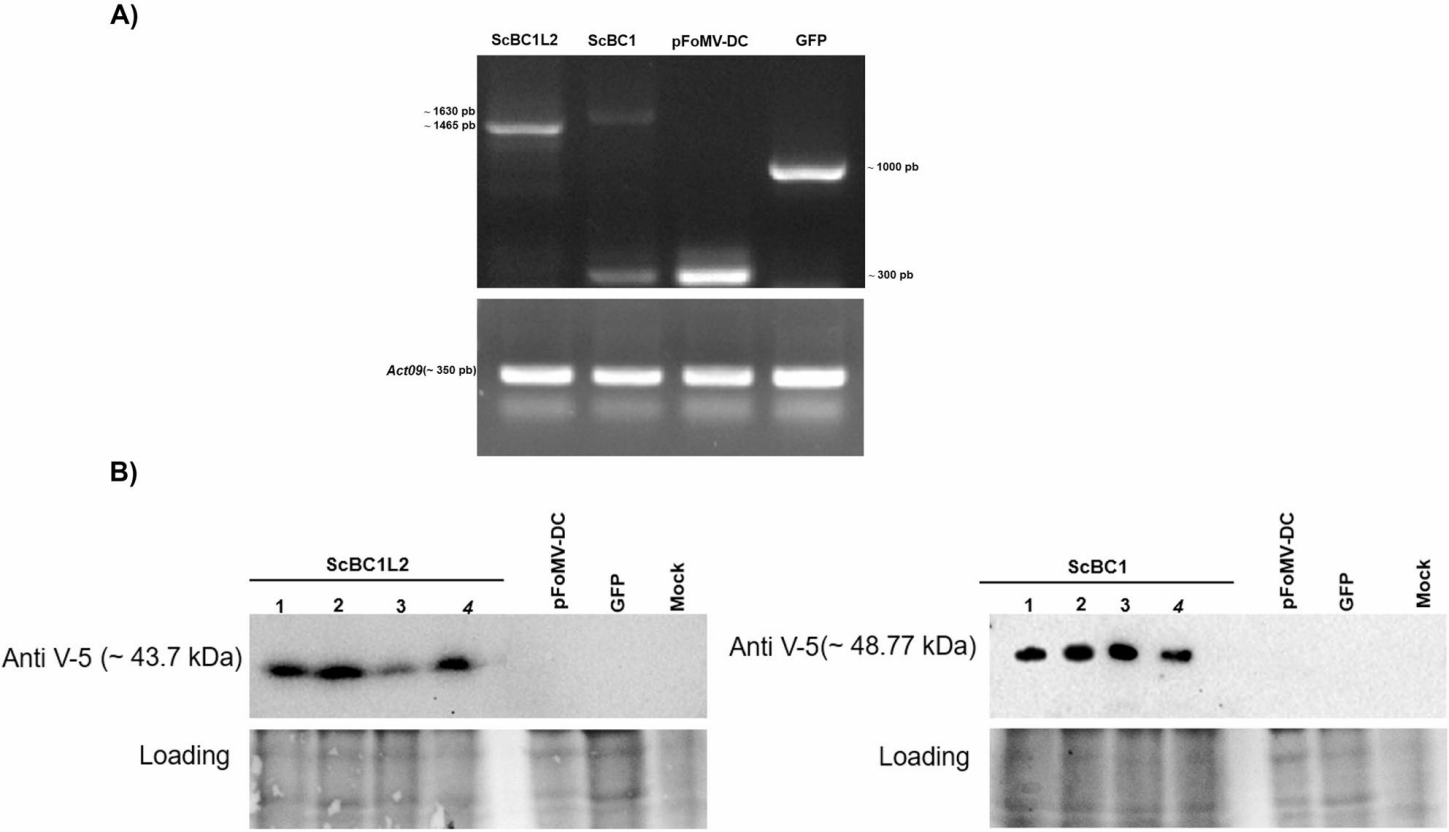
Overexpression of *ScBC1* and *ScBC1L2* via FoMV-VOX in *Nicotiana benthamiana *leaves. **A** RT-PCR analysis of FoMV-VOX constructs: *ScBC1L2* (1,465 bp), *ScBC1* (1,630 bp), *pFoMV-DC* empty vector (300 bp), and *GFP* (1,000 bp) expression in *N. benthamiana*. *NbACT09* was used as the internal constitutive control. **B** Western blot analysis using anti-V5 antibody to detect the expression of the ScBC1 and ScBC1L2 proteins. Upper panels show immunodetection with anti-V5 antibody; lower panels show total protein loading controls. Samples leave agroinfiltrated with FoMV-VOX-ScBC1, FoMV-VOX-ScBC1L2, and controls: pFoMV-DC (empty vector), FoMV-VOX-GFP, and Mock (water-inoculated)

To further investigate the function of *ScBC1* and *ScBC1L2*, FOMV-VOX-ScBC1 and FOMV-VOX-ScBC1L2 construction (Figure S1A and B) were infiltrated separately into *N. benthamiana* leaves. RT-PCR and Western blot analyses confirmed the heterologous expression of ScBC1 and ScBC1L2 proteins (Fig. 4). The FOMV-VOX-GFP construct was used as a control, and GFP protein expression was validated by RT-PCR, fluorescence microscopy, and immunoblot analyses (Figure S4).

**Fig. 5:**
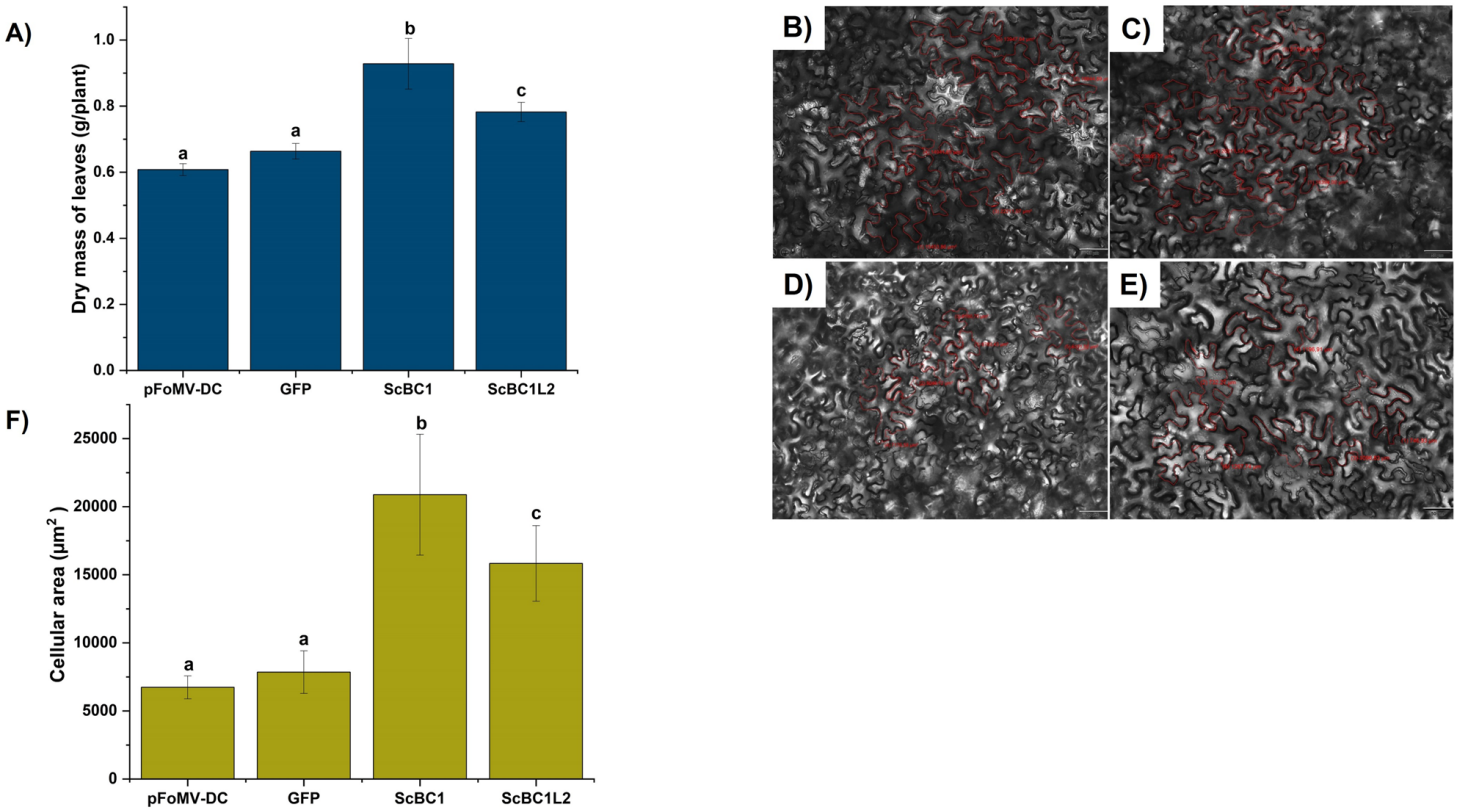
Effect of ScBC1 and ScBC1L2 overexpression via FoMV-VOX on leaf biomass and cell size. **A** Mean dry leaf mass (grams) of *N. benthamiana* leaves agroinfiltrated with FoMV-VOX constructs. (b–e) Brightfield microscopy images of the abaxial epidermis in leaves infiltrated with: **B** FoMV-VOX-ScBC1L2, **C** FoMV-VOX-ScBC1, **D** pFoMV-DC (empty vector), and (**E**) FoMV-VOX-GFP. Images captured at 50 ms exposure and 20× magnification; scale bar = 50 μm). **F** Mean cell area quantified from abaxial epidermal cells of the agroinfiltrated leaves. Different letters indicate statistically significant differences according to one-way ANOVA following Tukey’s test (*p* < 0.05)

The expression of ScBC1 and ScBC1L2 proteins in agroinfiltrated leaves resulted in dry weights of 0.92 ± 0.07 and 0.78 ± 0.03 g/plant, respectively. These values were significantly higher than those observed in the controls empty vector pFoMV-DC (0.60 ± 0.01 g/plant) and pFoMV-GFP (0.66 ± 0.02 g/plant) (Fig. 5). Furthermore, the heterologous expression of ScBC1 resulted in the highest dry weight among the samples analyzed (Fig. 5A). These findings suggest that ScBC1L2 and, more notably, ScBC1, play a significant role in biomass development.

Several studies suggest that COBRA proteins play functions in regulating cellulose deposition and cell wall organization, potentially guiding expansion in the plant cell wall [20, 21, 63]. To investigate the effects of ScBC1L2 and ScBC1 overexpression on plant tissue development, cell area was quantified for 220 cells from each sample group (Fig. 5B-F). The cell size in leaves agroinfiltrated with constructs expressing ScBC1 (mean 20880 ± 4438 μm²) and ScBC1L2 (mean 15836 ± 2776 μm²) was significantly larger than that in leaves infiltrated with the control constructs pFoMV-DC (mean 6731 ± 847 μm²) and pFoMV-GFP (mean 7848 ± 1564 μm²) (Fig. 5F).

Potential changes in cellulose content and other cell wall polymers associated with ScBC1L2 and ScBC1 protein expression were investigated in agroinfiltrated samples through determination of their chemical composition (Figure S5). However, no significant differences were detected in the levels of arabinose, rhamnose, mannose, galactose, or glucose, as well as in the amounts of matrix polysaccharides (pectic substances and hemicellulose) and glucose derived from crystalline cellulose in the cell wall. (Figure S5A, S5B and S5C) in *N. benthamiana* leaves expressing ScBC1L2 and ScBC1 compared to the wild-type, GFP, and *pFoMV-DC* controls. Together, these results suggest that the phenotypic alterations, including increased biomass and cell area, observed in ScBC1- and ScBC1L2-expressing agroinfiltrated *N. benthamiana* leaves (Fig. 5) were not associated with significant changes in the overall cell wall chemical composition of this plant.

In order to analyze FOMV-VOX-ScBC1 and FOMV-VOX-ScBC1L2 constructions in maize, an important grass model plant, *N. benthamiana* leaves agroinfiltrated were used as inoculum to achieve heterologous expression of *ScBC1* and *ScBC1L2* in maize. Unfortunately, no transgene expression was detected in the inoculated maize plants (data not shown).

### VIGS silencing of *ScBC1* and *ScBC1L2* maize orthologs

To complement the functional characterization of the *ScBC1* and *ScBC1L2* grass subclades in cell wall structure, and to address potential limitations of *N. benthamiana* as a heterologous expression model, a VIGS assay was performed in maize targeting *ZmBK2* and *ZmBK2L3*, the respective orthologs of *ScBC1* and *ScBC1L2* (Fig. 2). For this approach, FoMV-based VIGS constructs (FoMV-VIGS-ScBC1 and FoMV-VIGS-ScBC1L2) (Figure S1C and D) were engineered to carry antisense fragments from the coding sequences of *ScBC1* and *ScBC1L2*, which share 94.01% and 93.66% nucleotide identity with *ZmBK2* and *ZmBK2L3*, respectively. Leaves of *N. benthamiana* expressing the VIGS constructions were used as inoculum to infect maize leaves (Figure S6).

**Fig. 6:**
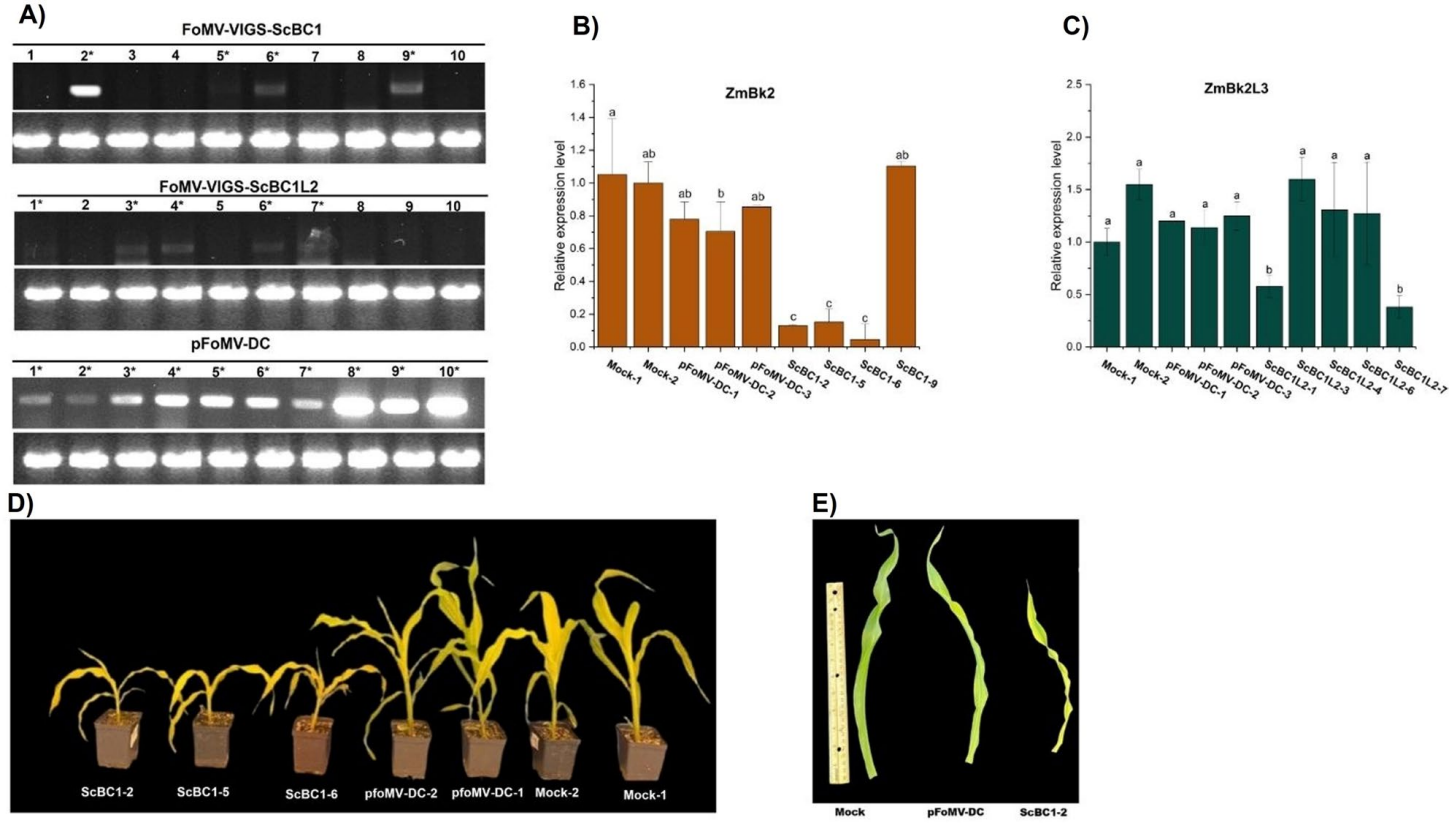
Molecular validation and phenotypic characterization of *ZmBK*-silenced maize plants. **A** RT-PCR amplification from FoMV-VIGS-ScBC1 (572 bp), FoMV-VIGS-ScBC1L2 (571 bp), and the control vector pFoMV-DC (300 bp) in maize leaves inoculated with the FoMV-VIGS constructs. *ZmACT01* was used as a constitutive reference gene. **B** Quantitative RT-PCR analysis of *ZmBK2* and (**C**) *ZmBK2L3* gene expression in maize plants infected with FoMV-VIGS-ScBC1, FoMV-VIGS-ScBC1L2, pFoMV-DC (empty vector), and Mock (buffer-infiltrated) controls. Gene expression calculated by the 2^−ΔΔCt^ method using *Zmβ-tubulin* as internal reference gene and Mock-2 as the reference sample. **D** Plant height and (**E**) leaf morphology of plants inoculated with FoMV-VIGS-ScBC1 compared to pFoMV-DC empty vector and Mock controls. Bars represent the standard deviation of three biological replicates. Different uppercase letters indicate statistically significant differences among treatments (Tukey’s test, *p* < 0.05)

The expression of antisense fragments of *ScBC1* and *ScBC1L2* in maize was confirmed by RT-PCR amplification (Fig. 6). Among the ten plants inoculated with each construct, four plants showed the expected amplification product for the *ScBC1* antisense fragment, while five exhibited amplification products for the *ScBC1L2* (Fig. 6A). These individual plants were selected for quantitative expression analysis by RT-qPCR to assess the silencing efficiency of endogenous maize *ZmBK2* and *ZmBK2L3* (Fig. 6B and C). Transcript quantification analysis in maize plants expressing the *ScBC1* antisense fragment indicated that three plants (ScBC1-2, ScBC1-5, and ScBC1-6) exhibited a significant reduction in *ZmBK2* expression levels compared to controls. In addition, among the plants expressing *ScBC1L2* antisense fragments, two individuals (ScBC1L2-1 and ScBC1L2-7) showed significant reduced expression of the *ZmBK2L3* gene relative to controls (Fig. 6C).

Maize plants silenced for *ZmBK2* and *ZmBK2L3* via FoMV-VIGS were phenotypically evaluated for plant height, leaf area, shoot dry mass, as well as total and crystalline glucose content. Plants with *ZmBK2* silencing had significantly lower values for all parameters analyzed compared to controls, including stunted growth and reduced cellulose accumulation (Fig. 6D and Figure S7). In contrast, *ZmBK2L3*-silenced plants did not exhibit significant phenotypic alterations (Figure S7).

### Quantification of cell area in VIGS-silenced maize plants

**Fig. 7:**
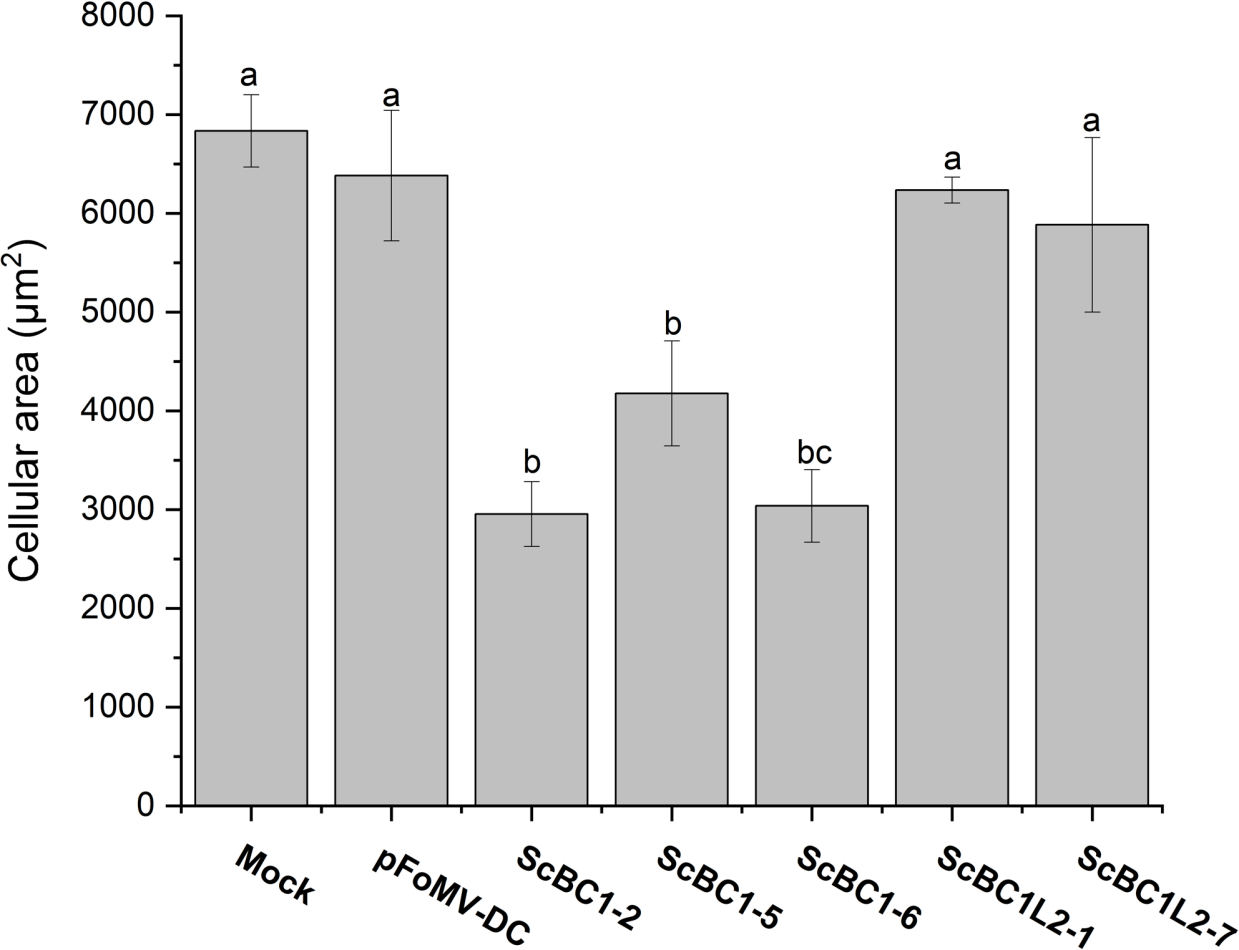
Cell area analysis of *ZmBk2* and *ZmBk2L3 *silenced maize leaves. Quantification of mean epidermal cell area. Different letters indicate statistically significant differences among treatments, as determined by one-way ANOVA followed by Tukey’s test (*p* < 0.05). Error bars represent standard deviations

To investigate whether silencing of *ZmBK2* and *ZmBK2L3* in maize results in phenotypic effects related to cell area observed in transient *ScBC1* and ScBC1L2 expression in *N. benthamiana* (Fig. 5F), we analyzed the leaf epidermal cell size using electron microscopy analysis. All *ZmBK2*-silenced maize plants showed a significant reduction in cell size, with decreases of approximately 50% compared to the controls (mock and empty vector). In contrast, *ZmBK2L3*-silenced plants (*ScBC1L2-1* and *ScBC1L2-7*) did not show significant changes in cell area compared to the controls (Fig. 7). No significant alterations in cell area were observed in ZmBK2L3-silenced maize plants (ScBC1L2-1 and ScBC1L2-7) compared to the controls (Fig. 7).

### Expression analysis of cell wall genes in *ZmBK2* VIGS-silenced maize plants.

Previous studies have demonstrated a strong correlation between the expression of *ZmBK2* and that of secondary cell wall *CesA* genes (*ZmCesA10*, *ZmCesA11*, and *ZmCesA12*) [15]. In the present study, *ZmBK2*-silenced plants exhibited a significant reduction in the transcript levels of *ZmCesA10* and *ZmCesA11*, supporting a disruption in secondary cell wall biosynthesis. *ZmCesA12* expression was also reduced, however, not statistically significant was observed (*p* = 0.1668731) (Figure S8). Altogether, the observed phenotypic alterations are consistent with the reduced *ZmBK2* transcript levels, confirming the effectiveness of gene silencing.

**Fig. 8:**
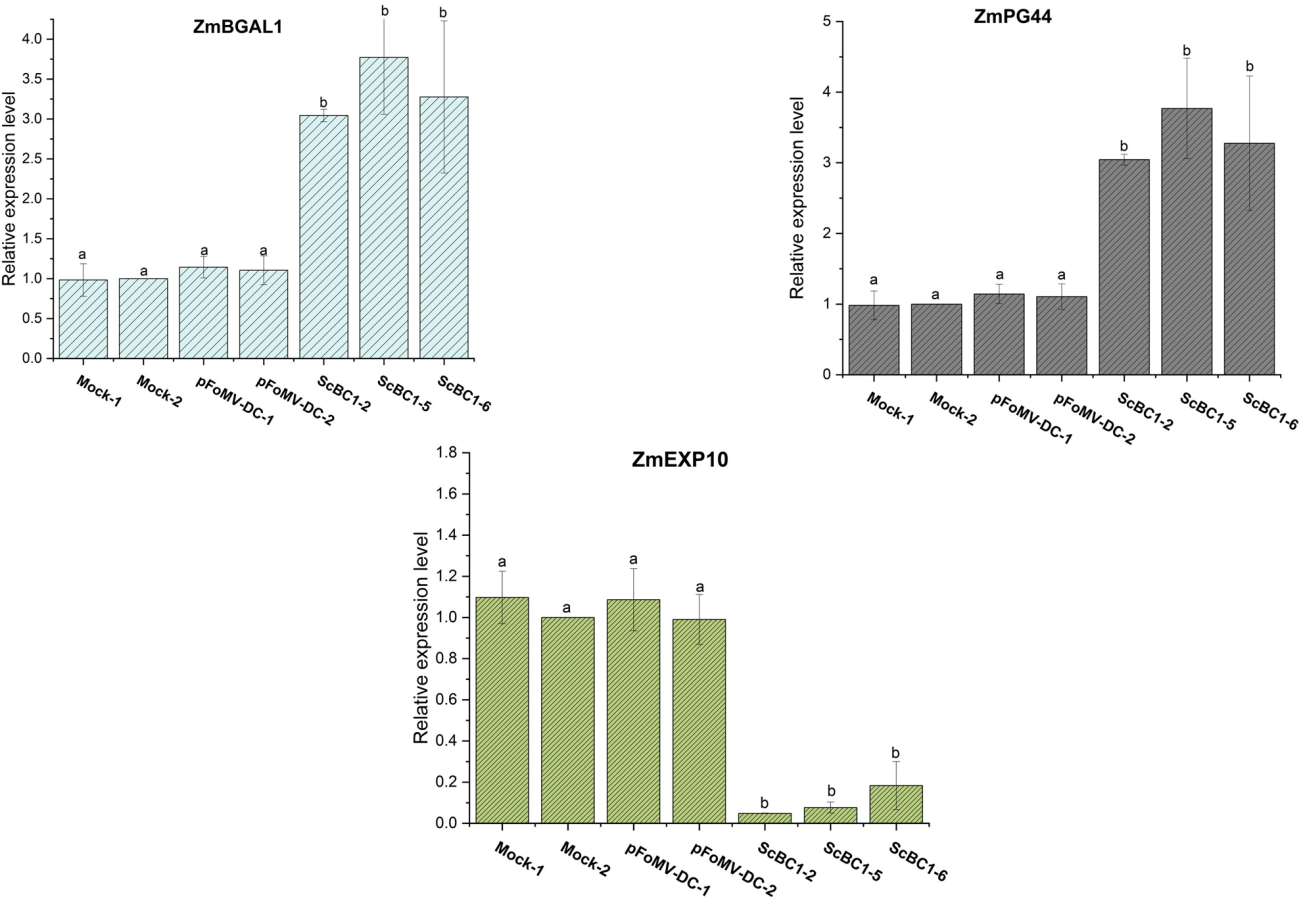
Impact of *ZmBK2 *silenced maize leaves in cell wall loosening genes. Relative transcript levels of *ZmBGAL1*, *ZmPL44*, and *ZmEXP10* in maize leaves silenced for *ZmBK2* using the FoMV-VIGS-ScBC1 construct (ScBC1-2, ScBC1-5, and ScBC1-6), compared to control plants inoculated with pFoMV-DC (empty vector) and Mock (water-inoculated). Relative gene expression calculated using the 2^−ΔΔCt^ method using *Zmβ-tubulin* as the internal reference gene and Mock-2 as the reference sample. Error bars indicate standard deviations, and different letters denote statistically significant differences among treatments (one-way ANOVA followed by Tukey’s test, *p* < 0.05)

Silencing of COBRA family genes has been shown to affect not only the expression of *CesA* genes but also other cell wall-modifying genes, including *β-galactosidases* (*BGALs*), *polygalacturonases* (*PLs*), and *expansins* (*EXPs*), as previously reported in tomato fruits [21]. To investigate whether *ZmBK2* silencing induces similar transcriptional responses in maize, we conducted RT–qPCR analyses to quantify the expression of *ZmBGAL1* and *ZmPG44*, which have been previously characterized [52, 53]. Both genes showed significantly increased expression levels in VIGS plants compared to mock and empty vector (pFoMV-DC) controls (Fig. 8). In contrast, *ZmEXP10*, which encodes a maize expansin involved in cell wall loosening during cell wall expansion [54], was significantly downregulated in *ZmBK2*-silenced plants (Fig. 8), consistent with the reduced leaf epidermal cell size observed in these plants (Fig. 7).

## Discussion

This study identified sugarcane COBRA genes and analyzed the potential role of two of them in cell wall modification. A total of 50 putative COBRA genes were identified in the sugarcane genome (R570) through comparative phylogenetic analysis (Table S1). The R570 cultivar, with its highly complex 10 Gb genome, contains approximately 12 copies of each chromosome. This high level of genomic redundancy, reflected in identical sequences across chromosomes (JGI, 2024), was also observed in the COBRA gene family (Table S1 and Figure S2). However, among all the 50 putative COBRA sequences identified, only eleven were selected for the phylogeny analysis, representing unigenes for each clade containing related sugarcane sequences (Fig. 2 and Table S1). Of these, only five genes were expressed in the datasets analyzed in silico (SUCEST and RNA-seq), with *ScBC1* and *ScBC1L2* standing out as the most highly expressed in stem bark and internodes (Fig. 3 and S3).

In the phylogenetic analysis, *ScBC1* clustered into Group I with *ZmBK2*, *OsBC1*, and *SbBC1* (Fig. 2), previously characterized genes in maize, rice, and sorghum, respectively [14, 60, 62]. Studies have shown that mutations in the *OsBC1* and *ZmBK2* genes led to a reduction in cellulose content, accompanied by an increase in lignin levels. These alterations impacted both mechanical resistance and elasticity, affecting the ability of cells and organs to maintain their proper shapes and positions [14, 62]. Additionally, *ZmBK2* is co-regulated with the *CesAs* genes involved in secondary cell wall formation in maize (*ZmCesA10*, *ZmCesA11*, and *ZmCesA12*), further supporting its role in this mechanism [15, 59]. Similarly, the *bc1* mutant in sorghum exhibited reduced mechanical strength, lower cellulose, and higher lignin content without, however, significantly affecting overall plant morphology. Transmission electron microscopy of the *bc1* mutant revealed a decrease in cell wall thickness in the sclerenchyma, usually extensively lignified [60]. On the other hand, *ScBC1L2* clustered into Group III with the maize gene *ZmBK2L3* (Fig. 2). The *bk2l3* mutant in maize exhibits reduced plant height, a significant deficiency in cellulose content, and a loss of anisotropic cell elongation in the root elongation zone [64]. These results suggest that *ScBC1* and *ScBC1L2* could play conserved roles in secondary and primary cell wall formation, respectively.

RT-qPCR analysis of different sugarcane organs (fifth internode of 1-year-old plants, young stems, and leaves from 3-month-old plants) in two non-commercial sugarcane hybrids (H89 and H140) revealed that *ScBC1* and *ScBC1L2* expression levels were significantly higher in young stems compared to internodes and leaves (Fig. 3B and C). The two sugarcane hybrids, previously characterized, were selected for this study due to their contrasting chemical composition and bagasse digestibility [36–38]. The H89 hybrid has been reported to exhibit lower lignin content, higher glucan levels in the internode region, and consequently, greater enzymatic saccharification efficiency. In contrast, H140 displays higher lignin content, lower glucan levels, and reduced enzymatic saccharification efficiency [36, 65]. *ScBC1* exhibited higher expression in the young stems of H140, the high-lignin-content hybrid, whereas *ScBC1L2* was more highly expressed in H89 (Fig. 3B and C). This finding indicates that both genes are predominantly expressed in tissues undergoing active growth. However, they exhibit preferential recruitment patterns that may vary among cultivars, possibly influenced by differences in lignin content, supporting the hypothesis that these genes could play hierarchical roles in the formation of primary and secondary cell walls.

In the current study, the cellulose crystallinity index (CI) was determined for the rind, a tissue with higher lignin content [37], from the fifth internode of both hybrids (Fig. 3D). The results indicate that H89 exhibits a higher CI in the rind compared to H140, suggesting a balance between lignin and cellulose crystallinity to maintain culm structure and mechanical stability. The higher CI in H89 may be associated with the higher expression of *ScBC1* in the internode compared to *ScBC1L2*, indicating a possible role of *ScBC1* in regulating crystalline cellulose content in this tissue. Previous studies have shown that mutations in *OsBC1*, a ortholog of *ScBC1*, is associated with reduced crystalline cellulose content [62].

Heterologous transient expression of ScBC1 and ScBC1L2 proteins in *N. benthamiana* via agroinfiltration led to a significant increase in leaf epidermal cell size and dry mass accumulation compared to controls, with ScBC1 inducing the most pronounced effect (Fig. 5). In line with these results, VIGS-mediated silencing of the endogenous *ZmBk2* gene, a ScBC1 ortholog, in maize resulted in a significant reduction of epidermal cell area (Fig. 7), alongside phenotypes characteristic of *bk2* maize mutants [62], including decreased plant height, reduced cellulose content (Figure S7). In contrast, plants silenced for the *ZmBK2L3*, ortholog of *ScBC1L2*, did not exhibit significant phenotypic changes (Figure S7), including no differences in dry mass or cell area, unlike the effects observed following transient expression of the sugarcane *ScBC1L2* (Fig. 5). These results may be attributed to insufficient transcript suppression levels (Fig. 6).

Members of the COBRA family may serve as essential regulators of cell expansion [8, 19]. In Arabidopsis, the *cob1* mutant showed abnormal radial expansion of root cells under restrictive conditions compared with the wild type [19]. Similarly, the *atcobl10* mutant resulted in abnormal cell wall organization in pollen tubes during growth [8]. Notably, the overexpression of *ClCOBL1* from the conifer *Cunninghamia lanceolata* in tobacco plants led to short but swollen corolla tubes due to a faster radial expansion of cells compared to longitudinal growth [20]. In addition, the knockout of *OsBC1L4*, member of the same *ScBC1–ZmBK2* clade, leads to abnormal cell expansion and decreased cellulose content [16]. These findings support a conserved role for the *ScBC1–ZmBK2* subclade in the regulation of cell size.

Effective regulation of cell expansion orientation requires the cell’s ability to control the deposition and spatial organization of cellulose microfibrils [10]. Although no significant differences in cellulose content were observed in *N. benthamiana* leaves transiently expressing *ScBC1* or *ScBC1L2* (Figure S6), this could reflect the limitations of a heterologous expression that already contains cellulose and has an endogenous cell wall machinery that could partly restrict sugarcane COBRA protein activities. In contrast, and as expected, silencing *ZmBk2* in maize resulted in a significant reduction in both total and crystalline cellulose content (Figure S7A and B), consistent with the cellulose deficiencies previously reported in *bk2* maize mutants [62] and *bc1* sorghum mutants [60]. In line with these results, RT-qPCR analysis of *ZmBk2*-silenced plants revealed downregulation of secondary cell wall *CesA* genes (*ZmCesA10* and *ZmCesA11*), except for *ZmCesA12* (Figure S8). This pattern of co-regulation is consistent with previous observations from transcriptome analyses of *bk2* mutant lines [15].

Beyond *CesA* genes, the downregulation of *COBRA* genes may also affect the expression of transcripts encoding cell wall proteins involved in remodeling and degradation processes, typically associated with developmental stages such as maturation and senescence (21, 66). Silencing of *ZmBk2* resulted in the transcript upregulation of the previously characterized maize cell wall–modifying enzymes β-galactosidase 1 (*ZmBGAL1*) and polygalacturonase (*ZmPG44*) (Fig. 8). *ZmBGAL1* is typically involved in the removal of galactose residues from arabinogalactan and pectin polymers [52], whereas *ZmPG44* hydrolyzes glycosidic bonds between galacturonic acid units within pectin chains [53]. Similar transcriptional responses were observed in transgenic tomato fruits with silenced *COBRA*-like genes [21]. The upregulation of β-galactosidase and polygalacturonase genes in *COBRA*-silenced plants may reflect a compensatory remodeling response, in which the degradation of pectins or hemicelluloses would facilitate the reorganization of the cell wall architecture in reaction to reduced cellulose content and crystallinity. Interestingly, *OsBC1L4* knockout plants exhibited the opposite phenotype, with increased pectin levels [16], suggesting that those responses are not conserved among homologous genes within the *ScBC1-ZmBK2* clade, but may instead reflect species-specific compensatory mechanisms to preserve cell wall integrity.

*ZmBk2*-silenced maize plants also exhibited significantly reduced expression of *ZmEXP10* [54] compared to controls (Fig. 8). Expansins are key regulators of cell expansion, functioning by loosening the cellulose–hemicellulose network [54]. The downregulation of *ZmEXP10* may impair normal cell wall extensibility, consistent with the reduced epidermal cell size observed in *ZmBk2*-silenced plants (Fig. 7). In line with these findings, transcriptomic analysis of *cob5* mutants in *Arabidopsis* revealed significant downregulation of six expansin genes (66), suggesting that additional members of the maize expansin gene family may also be affected by *ZmBK2* silencing.

## Conclusions

Together, these findings support a conserved role for the *ScBC1–ZmBK2* clade in cellulose deposition and cell expansion and highlight *ScBC1* as a promising target for biotechnological strategies aimed at enhancing biomass quality. These results pave the way for further functional validation and the development of genetically improved energy crops.

## Supplementary Information

Supplementary Material 1.

## Data Availability

The genomic and transcriptomic datasets supporting this study are available in the Phytozome ([https://phytozome-next.jgi.doe.gov/](https://phytozome-next.jgi.doe.gov)) and NCBI ([https://www.ncbi.nlm.nih.gov/](https://www.ncbi.nlm.nih.gov)) repositories. Accession numbers are provided in Supporting Information Table S1. All other data supporting the findings of this study are included in the article and its supplementary materials.
