# Supplementary material for: Expression of sugarcane COBRA-Like genes, ScBC1 and ScBC1L2, increases plant biomass
Source: BMC Plant Biol. 2025 Dec 15;26:97. doi: 10.1186/s12870-025-07910-y (PMC12822275; doi:10.1186/s12870-025-07910-y)

**Supplementary Materials**

Expression of sugarcane COBRA-Like genes, *ScBC1 and ScBC1L2*, increases plant biomass

**Supplementary Table S1:**

**Supplementary Table S1:** *COBRA* gene codes from the genomes of *Saccharum spp., Zea mays, Sorghum bicolor*, *Oryza sativa*, and *Arabidopsis thaliana*. *Saccharum* sequences are shown in different colors to represent distinct groups of related sequences.

| *Sugarcane* (*Saccharum spp.*) | | | *Zea mays* | | *Sorghum bicolor* | | *Oryza sativa* | | *Arabidopsis thaliana* | |
| --- | --- | --- | --- | --- | --- | --- | --- | --- | --- | --- |
| Phytozome Gene Identifier | **Sequence name** | **Chromosome Location** | **Phytozome Gene Identifier** | **Sequence name** | **Phytozome Gene Identifier** | **Sequence name** | **Phytozome Gene Identifier** | **Sequence name** | **Phytozome Gene Identifier** | **Sequence name** |
| SoffiXsponR570.01Bg309400.1 | ScBC1 | Chr1B:67280589..67284194 | Zm00001d047276_T001 | ZmBk2 | Sobic.001G336600.1 | SbBC1L4 | Os03g30260.1 | OsBC1L2 | AT5G60920.1 | AtCOB |
| SoffiXsponR570.01Fg138200.1 |  | Chr1F:38775095..38777631 | Zm00001d022082_T001 | ZmBk2L7 | Sobic.001G086200.1 | SbBC1L2 | Os07g41320.1 | OsBC1L7 | AT3G02210.1 | AtCOBL1 |
| SoffiXsponR570.01Eg252300.1 |  | Chr1E:51478705..51481077 | Zm00001d021661_T001 | ZmBk2L5 | Sobic.001G336700.1 | SbBC1 | Os03g30250.1 | OsBC1 | AT3G29810.1 | AtCOBL2/3 |
| SoffiXsponR570.01Dg198000.1 |  | Chr1D:51959626..51962041 | Zm00001d013256_T001 | ZmBk2L4 | Sobic.001G399900.1 | SbBC1L7 | Os03g18910.1 | OsBC1L1 | AT5G15630.1 | AtCOBL4 |
| SoffiXsponR570.01Cg187000.1 |  | Chr1C:52108385..52111851 | Zm00001d022081_T001 | ZmBk2L6 | Sobic.002G368100.1 | SbBC1L3 | Os05g32110.1 | OsBC1L3 | AT5G60950.1 | AtCOBL5 |
| SoffiXsponR570.01Ag339200.1 |  | Chr1A:88610997..88613467 | Zm00001d007208_T001 | ZmBk2L8 | Sobic.002G368300.1 | SbBC1L1 | Os07g41310.1 | OsBC1L6 | AT1G09790.1 | AtCOLB6 |
| SoffiXsponR570.8_5Ag207200.1 | ScBC1L1 | Chr8_5A:61084925..61088676 | Zm00001d028826_T001 | ZmBk2L1 | Sobic.002G427400.1 | SbBC1L8 | Os03g22810.1 | OsBC1L4 | AT4G16120.1 | AtCOBL7 |
| SoffiXsponR570.05Cg329000.1 |  | Chr5C:81655296..81657143 | Zm00001d002803_T001 | ZmBk2L9 | Sobic.006G163200.1 | SbBC1L6 | Os06g47110.1 | OsBC1L5 | AT3G16860.1 | AtCOBL8 |
| SoffiXsponR570.5_9Ag066600.1 |  | Chr5_9A:19314534..19317359 | Zm00001d034049_T001 | ZmBk2L3 | Sobic.001G086000.1 | SbBC1L5 | Os07g49080.1 | OsBC1L8 | AT5G49270.1 | AtCOBL9 |
| SoffiXsponR570.05Eg327000.2 |  | Chr5E:76705863..76708660 |  |  | Sobic.002G323200.1 | SbBC1L9 | Os10g35460.1 | OsBC1L9 | AT3G20580.1 | AtCOBL10 |
| SoffiXsponR570.05Fg261300.1 |  | Chr5F:69123116..69126002 |  |  |  |  | Os04g45700.1 | OsBC1L-p1 | AT4G27110.1 | AtCOBL11 |
| SoffiXsponR570.05Ag332900.1 |  | Chr5A:84180741..84182981 |  |  |  |  |  |  |  |  |
| SoffiXsponR570.05Bg325700.2 |  | Chr5B:82141573..82144459 |  |  |  |  |  |  |  |  |
| SoffiXsponR570.01Bg082800.1 | ScBC1L2 | Chr1B:11709689..11713292 |  |  |  |  |  |  |  |  |
| SoffiXsponR570.01Eg090700.1 |  | Chr1E:19254544..19258188 |  |  |  |  |  |  |  |  |
| SoffiXsponR570.01Ag089800.1 |  | Chr1A:19302928..19306547 |  |  |  |  |  |  |  |  |
| SoffiXsponR570.05Ag332800.1 | ScBC1L3 | Chr5A:84151007..84154911 |  |  |  |  |  |  |  |  |
| SoffiXsponR570.8_5Ag207100.1 |  | Chr8_5A:61051081..61055062 |  |  |  |  |  |  |  |  |
| SoffiXsponR570.05Fg261200.1 |  | Chr5F:69089966..69093692 |  |  |  |  |  |  |  |  |
| SoffiXsponR570.05Bg325600.1 |  | Chr5B:82108186..82112088 |  |  |  |  |  |  |  |  |
| SoffiXsponR570.05Cg328800.1 |  | Chr5C:81630906..81634682 |  |  |  |  |  |  |  |  |
| SoffiXsponR570.5_9Ag066700.1 |  | Chr5_9A:19326239..19330299 |  |  |  |  |  |  |  |  |
| SoffiXsponR570.01Fg138100.1 | ScBC1L4 | Chr1F:38770588..38775804 |  |  |  |  |  |  |  |  |
| SoffiXsponR570.01Dg197900.1 |  | Chr1D:51955947..51959601 |  |  |  |  |  |  |  |  |
| SoffiXsponR570.01Ag339100.1 |  | Chr1A:88607381..88610918 |  |  |  |  |  |  |  |  |
| SoffiXsponR570.01Cg187100.1 |  | Chr1C:52111648..52114270 |  |  |  |  |  |  |  |  |
| SoffiXsponR570.01Eg252200.2 |  | Chr1E:51465251..51478689 |  |  |  |  |  |  |  |  |
| SoffiXsponR570.01Bg309300.1 |  | Chr1B:67278055..67281638 |  |  |  |  |  |  |  |  |
| SoffiXsponR570.01Eg090600.1 | ScBC1L5 | Chr1E:19228626..19251213 |  |  |  |  |  |  |  |  |
| SoffiXsponR570.01Ag089700.1 |  | Chr1A:19285820..19302806 |  |  |  |  |  |  |  |  |
| SoffiXsponR570.01Ag089600.2 | ScBC1L5.1 | Chr1A:19246485..19249772 |  |  |  |  |  |  |  |  |
| SoffiXsponR570.01Eg090500.1 |  | Chr1E:19197453..19201831 |  |  |  |  |  |  |  |  |
| SoffiXsponR570.07Eg104900.1 | ScBC1L6 | Chr7E:34195082..34197500 |  |  |  |  |  |  |  |  |
| SoffiXsponR570.07Dg144700.1 |  | Chr7D:44852868..44855342 |  |  |  |  |  |  |  |  |
| SoffiXsponR570.7_10Ag262200.1 |  | Chr7_10A:74574600..74577113 |  |  |  |  |  |  |  |  |
| SoffiXsponR570.07Cg156700.1 |  | Chr7C:44303644..44306471 |  |  |  |  |  |  |  |  |
| SoffiXsponR570.07Ag144800.1 |  | Chr7A:47651466..47654322 |  |  |  |  |  |  |  |  |
| SoffiXsponR570.01Ag403500.1 | ScBC1L7 | Chr1A:102799259..102801524 |  |  |  |  |  |  |  |  |
| SoffiXsponR570.01Fg199300.1 |  | Chr1F:49883761..49886927 |  |  |  |  |  |  |  |  |
| SoffiXsponR570.01Cg250800.1 |  | Chr1C:65252089..65255262 |  |  |  |  |  |  |  |  |
| SoffiXsponR570.01Eg315300.1 |  | Chr1E:61317686..61320227 |  |  |  |  |  |  |  |  |
| SoffiXsponR570.01Dg257800.1 |  | Chr1D:62242764..62246081 |  |  |  |  |  |  |  |  |
| SoffiXsponR570.05Fg318800.1 | ScBC1L8 | Chr5F:79876028..79878158 |  |  |  |  |  |  |  |  |
| SoffiXsponR570.05Dg385200.1 |  | Chr5D:82929229..82931638 |  |  |  |  |  |  |  |  |
| SoffiXsponR570.05Bg384100.1 |  | Chr5B:93393479..93395676 |  |  |  |  |  |  |  |  |
| SoffiXsponR570.05Ag386500.1 |  | Chr5A:95476922..95479113 |  |  |  |  |  |  |  |  |
| SoffiXsponR570.05Cg385800.1 |  | Chr5C:92214593..92216925 |  |  |  |  |  |  |  |  |
| SoffiXsponR570.5_9Ag011600.1 |  | Chr5_9A:10804310..10806542 |  |  |  |  |  |  |  |  |
| SoffiXsponR570.05Fg213800.1 | ScBC1L9 | Chr5F:59969692..59972424 |  |  |  |  |  |  |  |  |
| SoffiXsponR570.05Dg286200.1 |  | Chr5D:67343710..67346398 |  |  |  |  |  |  |  |  |
| SoffiXsponR570.05Eg279100.1 |  | Chr5E:67705190..67707934 |  |  |  |  |  |  |  |  |
| SoffiXsponR570.05Cg282000.1 |  | Chr5C:72419157..72421901 |  |  |  |  |  |  |  |  |
| SoffiXsponR570.5_9Ag113200.1 |  | Chr5_9A:26892690..26895578 |  |  |  |  |  |  |  |  |
| SoffiXsponR570.05Bg277700.1 |  | Chr5B:72984451..72987175 |  |  |  |  |  |  |  |  |
| SoffiXsponR570.05Ag287200.1 |  | Chr5A:74859966..74862781 |  |  |  |  |  |  |  |  |

**Supplementary Table S2:**

**Supplementary Table S2:** Sequences of RT-qPCR primers.

| Primer name | Nucleotide sequence (5’–3’) |
| --- | --- |
| *ScBC1-F* | CCCATTGCTGCAAGAAAGAT |
| *ScBC1-R* | TTGCTGGGTCCTGGTTAAAG |
| *ScBC1L2-F* | GGTGACGATGAGCAACTACCA |
| *ScBC1L2-R* | TGGACCAGATCACCTCCTTC |
| *Rq_ZmBk2F* | TACTGTTGCTGCTCCTGGTG |
| *Rq_ZmBk2R* | CCCTGCCTACTGAAGTCTGC |
| *Rq_ZmBk2L3F* | GCTGCTCTGTTGGCTTATGC |
| *Rq_ZmBk2L3R* | TGCAGTGCACCTCATGGAAT |
| *ZmBGAL1d-F* | AAACACACACAGCAGCGAAC |
| *ZmBGAL1d-R* | ACCTGAACGAGGTTTGGTTG |
| *ZmPGL44-F* | CAAGGCCTCACCTTCTTGAG |
| *ZmPGL44-R* | GGTCTGAAGTCGGCAGAAAG |
| *ZmEXPA10-F* | TAAGGCTCCATCTGCTGCAT |
| *ZmEXPA10-R* | CAGCTGGACGTGATGAGATG |
| *ZmCesA10-F* | GTGTGCTGTTGCAGGAAATAC |
| *ZmCesA10-R* | CAGAGCATAGAGGACGAAGAATAG |
| *ZmCesA11-F* | GAGATGGTGAAGAATCTGGGTTAG |
| *ZmCesA11-R* | GCACAGTGTTTGCAGTGATTAG |
| *ZmCesA12-F* | CGAAACCATACCGGTTCAAATG |
| *ZmCesA12-R* | GAAGAGGGCGATGAAGAAGAG |
| *Zmβ-TUB-F* | CTACCTCACGGCATCTGCTATGT |
| *Zmβ-TUB-R* | GTCACACACACTCGACTTCACG |
| *ZmAct1-F* | AAGCTTGCCTACGTTGCCCTTGAT |
| *ZmAct1-R* | TGCTGAAAAGTGCTGAGAGAAGCCA |

**Supplementary Table S3:**

**Supplementary Table S3:** Nucleotide sequences of primers used for the isolation and cloning of the complete coding sequences of *ScBC1* and *ScBC1L2* into the FoMV viral vector for virus-mediated overexpression (VOX) in plants using the HiFi cloning system. Homology regions with the pFoMV-DC vector are highlighted and nucleotide sequences of the primers used in conventional PCR to confirm the expected bands for VOX and virus-induced gene silencing (VIGS) constructs.

| Gene | Nucleotide sequence (5’–3’) |
| --- | --- |
| ScBC1-F | ATGGGGCTCCGCGACT |
| ScBC1-R | TGCTACCAGGAGCAGCG |
| ScBC1L2-F | ATGTATAATTTTCAACAATTTCGGC |
| ScBC1L2-R | TGCATAAGCCAACAGAGCAG |
| ScBC1-VOX-F | **GAGAAACCCTTAGGAGAGTT**ATGGGGCTCCGCGACTCCTCC |
| ScBC1-VOX-R | CTAGGTACTGTCAAGACCGAGAAGGGGATTTGGGATTGGCTTCCCTGCTACCAGGAGCAGCGC |
| ScBC1L2-VOX-F | **GAGAAACCCTTAGGAGAGTT**ATGTATAATTTTCAACAATTTCGGCACATCGGCGCACC |
| ScBC1L2-VOX-R | CTAGGTACTGTCAAGACCGAGAAGGGGATTTGGGATTGGCTTCCCTGATAAGCCAACAGAG |
| FoMV-V5-R | **GTTGAGTGGGGCCCGTT**GTTCTAGGTACTGTCAAGACC |
| FoMV-MCS-VOX-F | ATAGCTCTGTGGTGTCTAAGCA |
| FoMV-MCS-VOX-R | GTCGGCATTTTGTGTTGC |
| FoMV-MCSI-VIGS-F | TCTGTACCGTACGATGAGCCC |
| FoMV-MCSI-VIGS-R | GCTGCGTTACTGTTAGGTCG |

**Supplementary Table S4:**

**Supplementary Table S4.** Correspondence between COBRA gene sequences (*ScBC1* and *ScBC1L1-9*) from *Saccharum* spp. selected for phylogenetic analysis and identified in the PHYTOZOME v11.0 database, and their homologous sequences in the SUCEST and RNA-seq datasets. The table presents sequence identity scores obtained from pairwise comparisons.

| Gene name | Gene ID PHYTOZOME | Gene ID SUCEST | % Identity (SUCEST) | Gene ID RNAseq | % Identity (RNAseq) |
| --- | --- | --- | --- | --- | --- |
| ScBC1 | SoffiXsponR570.01Bg309400.1 | SCCCST3002E04.g | 100 | Locus1370.10_Confidence_0.595_Length_1794 | 100 |
| ScBC1L1 | SoffiXsponR570.8_5Ag207200.1 | SCBFST3134F07.g | 99 | Locus30369.1_Confidence_1.000_Length_349 | 99 |
| ScBC1L2 | SoffiXsponR570.01Bg082800.1 |  |  | Locus1037.8_Confidence_0.233_Length_776 | 99 |
| ScBC1L3 | SoffiXsponR570.05Ag332800.1 | SCMCSD1062E01.g | 100 |  |  |
| ScBC1L4 | SoffiXsponR570.01Fg138100.1 |  |  |  |  |
| ScBC1L5 | SoffiXsponR570.01Eg090600.1 |  |  |  |  |
| ScBC1L15.1 | SoffiXsponR570.01Ag089600.2 | SCJFRT1061B08.g | 100 |  |  |
| ScBC1L6 | SoffiXsponR570.07Eg104900.1 |  |  |  |  |
| ScBC1L7 | SoffiXsponR570.01Ag403500.1 | SCEZLB1007H11.g | 100 | Locus11418.1_Confidence_1.000_Length_2186 | 100 |
| ScBC1L8 | SoffiXsponR570.05Fg318800.1 |  |  |  |  |
| ScBC1L9 | SoffiXsponR570.05Fg213800.1 |  |  |  |  |

**Supplementary Fig. S1**

**Fig. S1:** Complete DNA sequence constructs and corresponding maps: FoMV-VOX-ScBC1L2 (A) and FoMV-VOX-ScBC1 (B). The FoMV constructs were generated using the pFoMV-DC backbone and the full-length *ScBC1L2* and *ScBC1* cDNAs. Cloning region map of the pFoMV-V vector (C). The *Mlu*I restriction site, viral reading frames, promoter, and terminator regions are shown. Image generated using SnapGene Viewer version 6.2.1 (<https://www.snapgene.com/snapgene-viewer>). FoMV-VIGS-ScBC1L2 (D) and FoMV-VIGS-ScBC1 (E).

**A) FoMV-VOX-ScBC1L2** **plasmid construct map**


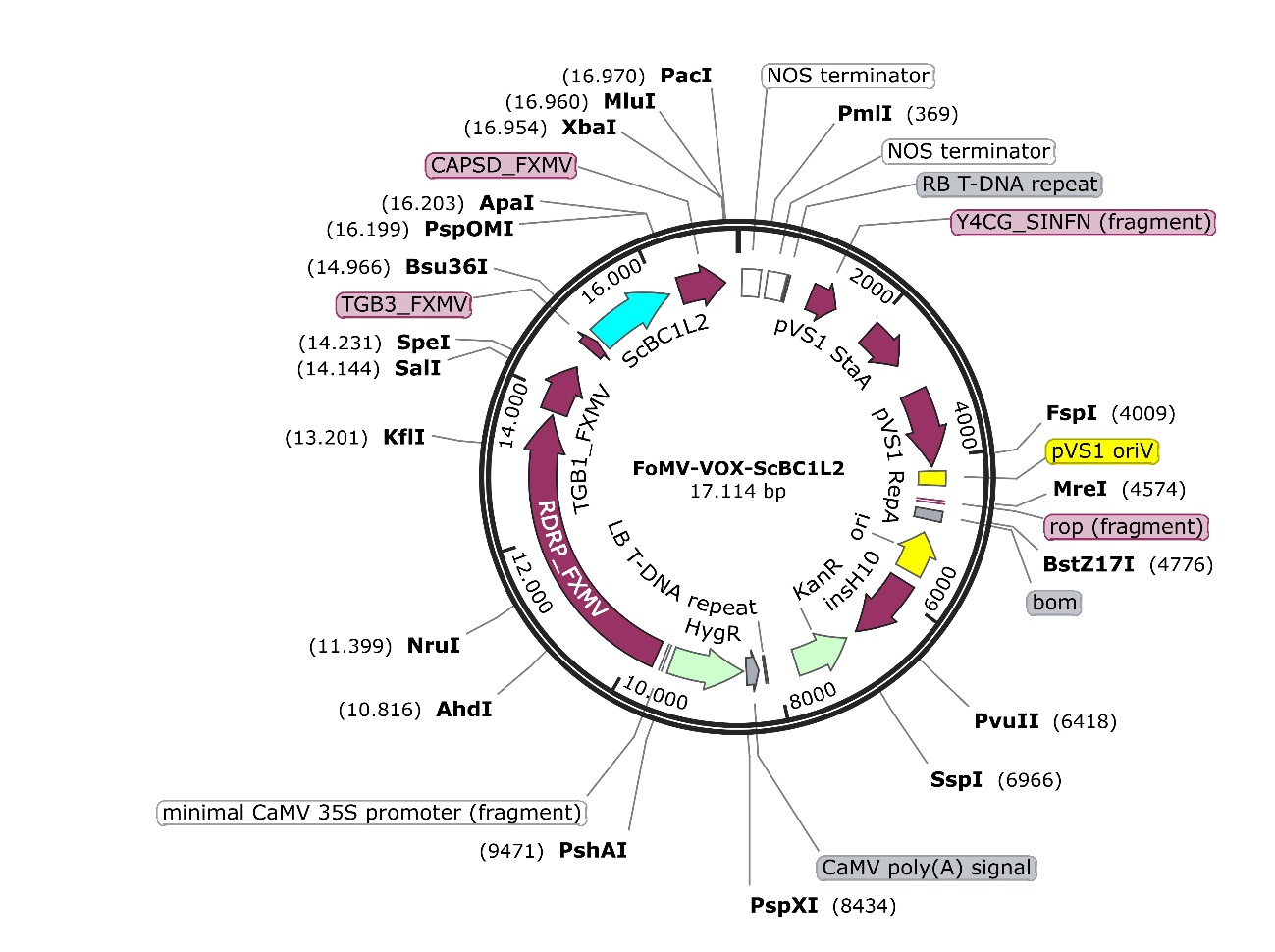


1. **FoMV-VOX-ScBC1 plasmid construct map**


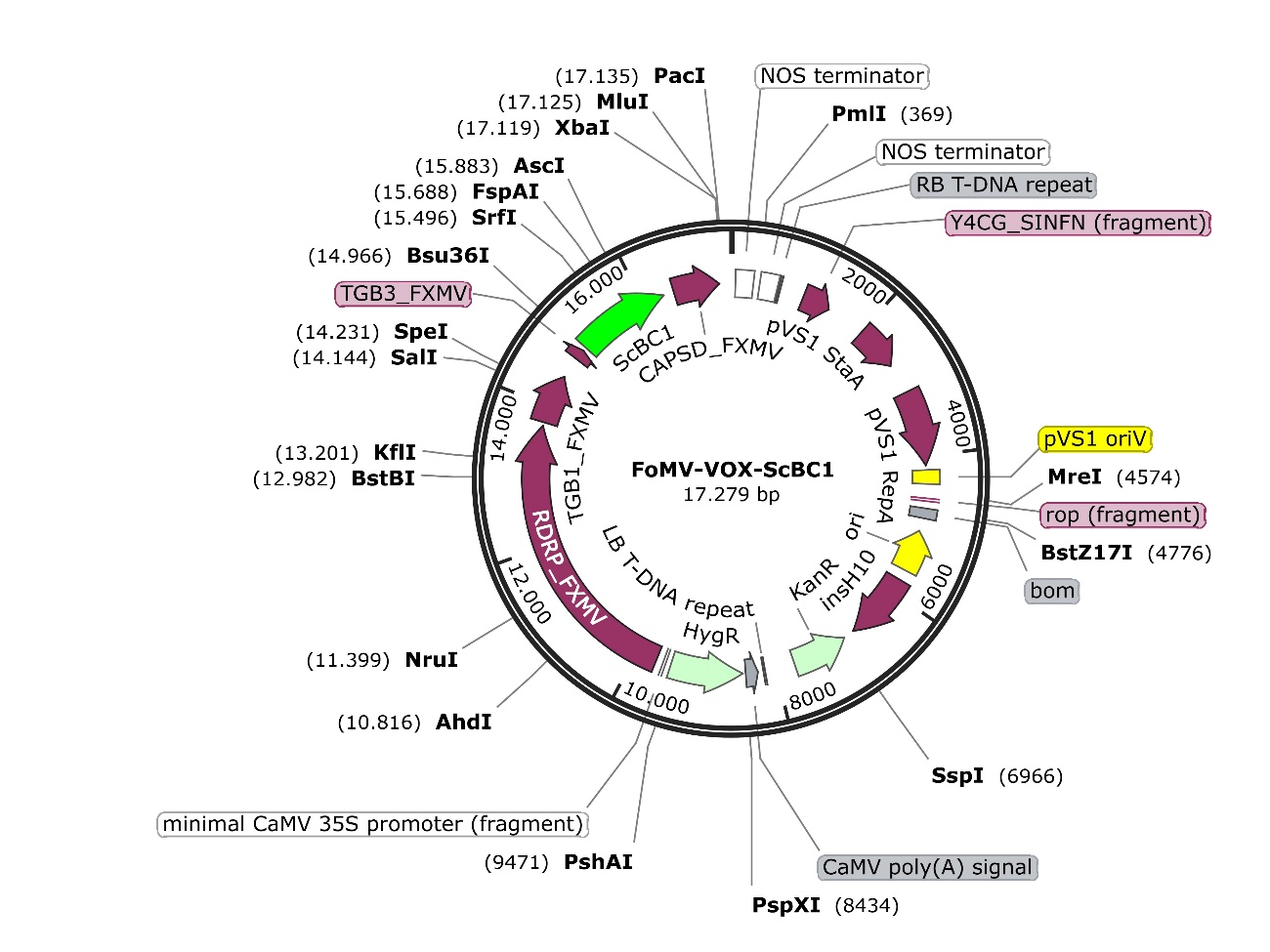


**C) FoMV-VIGS-ScBC1L2 plasmid construct map**


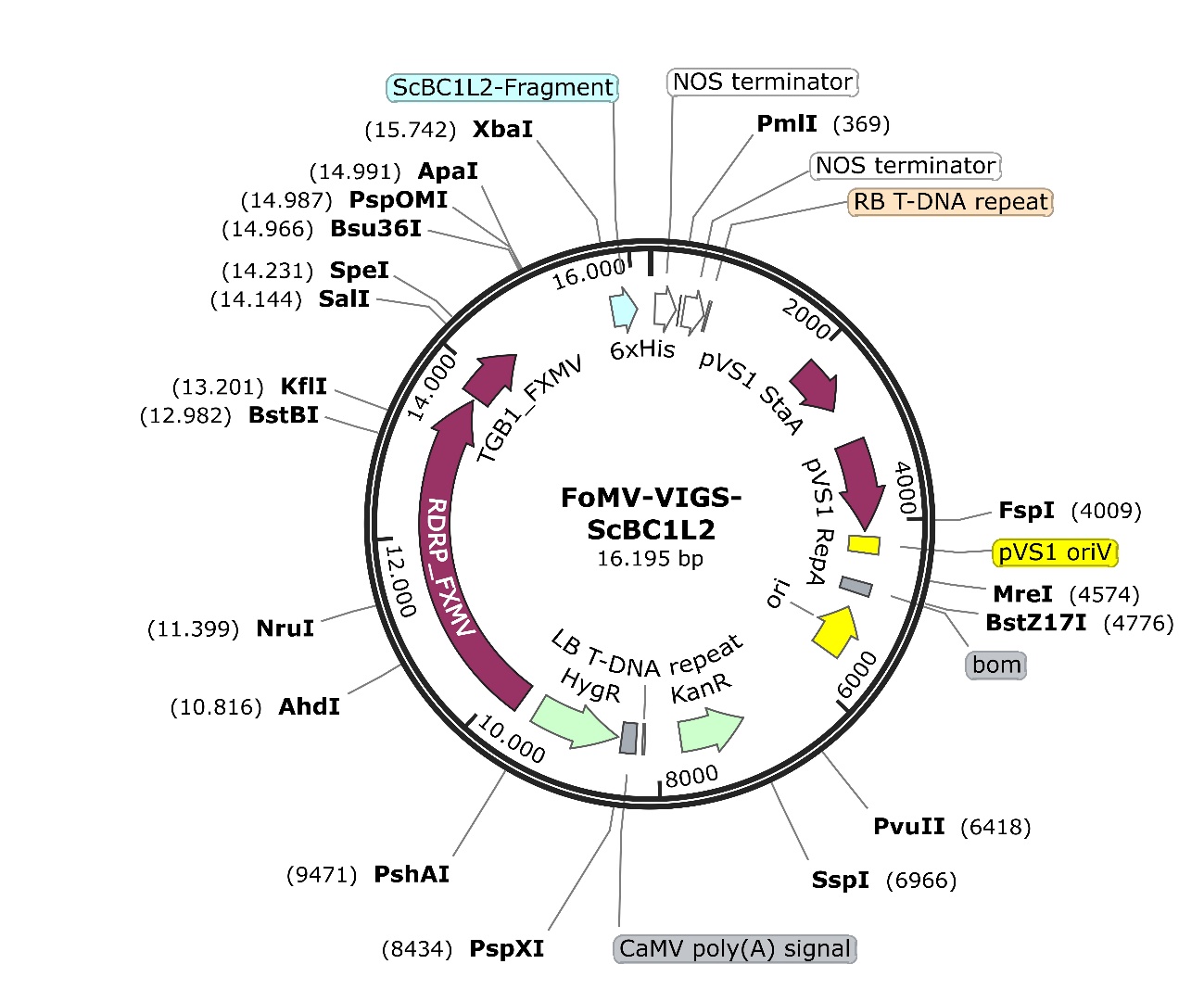


1. **FoMV-VIGS-ScBC1 plasmid construct map**


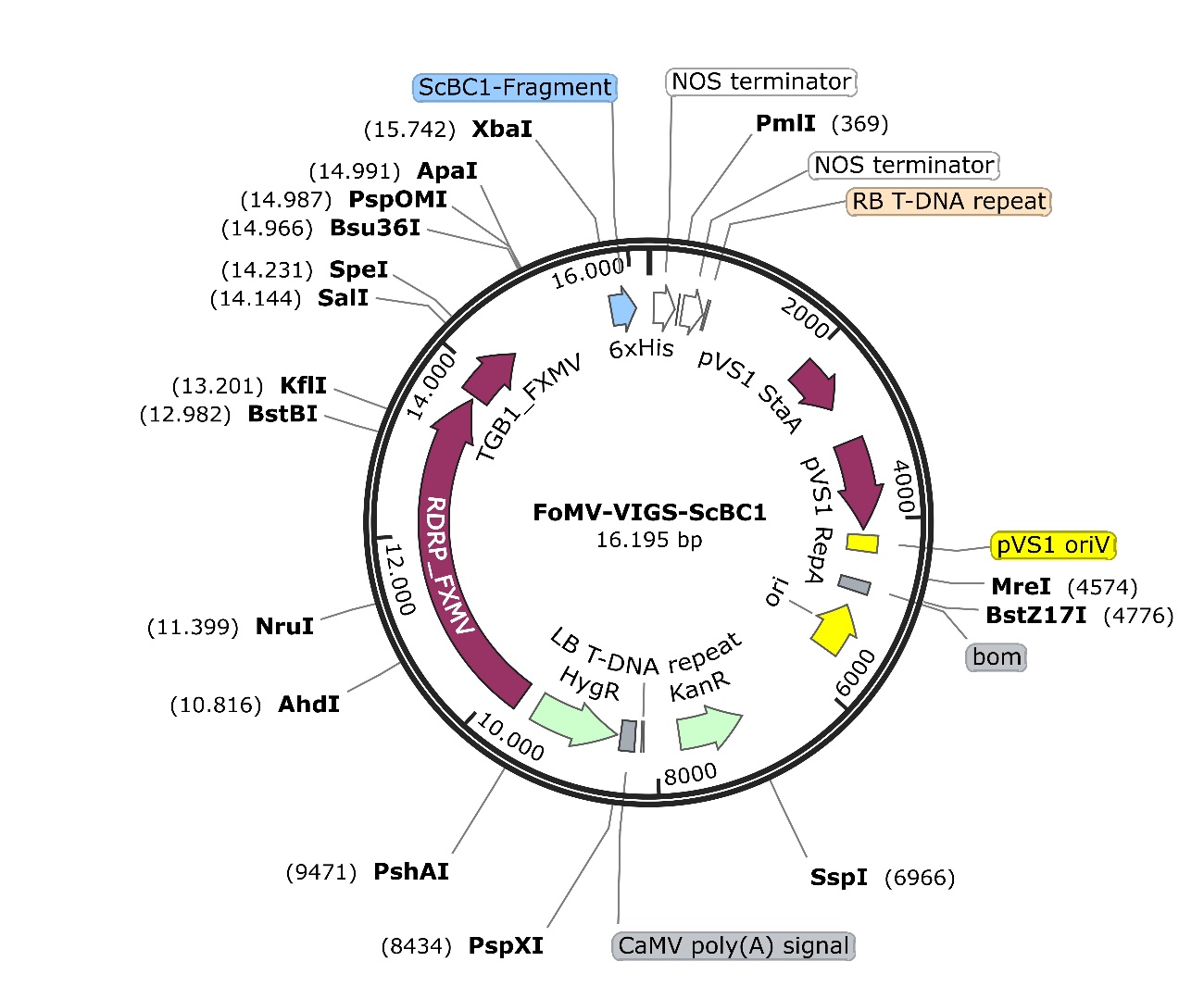


**Supplementary Fig. S2**

**Fig. S2:** The physical distribution of 11 putative *COBRA* genes across different chromosomes (1A, B, E, F; 5F; 7E; and 8_5A) was determined based on data retrieved from Phytozome (*Saccharum officinarum × spontaneum* R570 v2.1). Chromosomes were drawn to scale, with their respective numbers indicated below each ideogram. Genes belonging to different major clades are represented in distinct colors, following the same color scheme as in Figure 2.


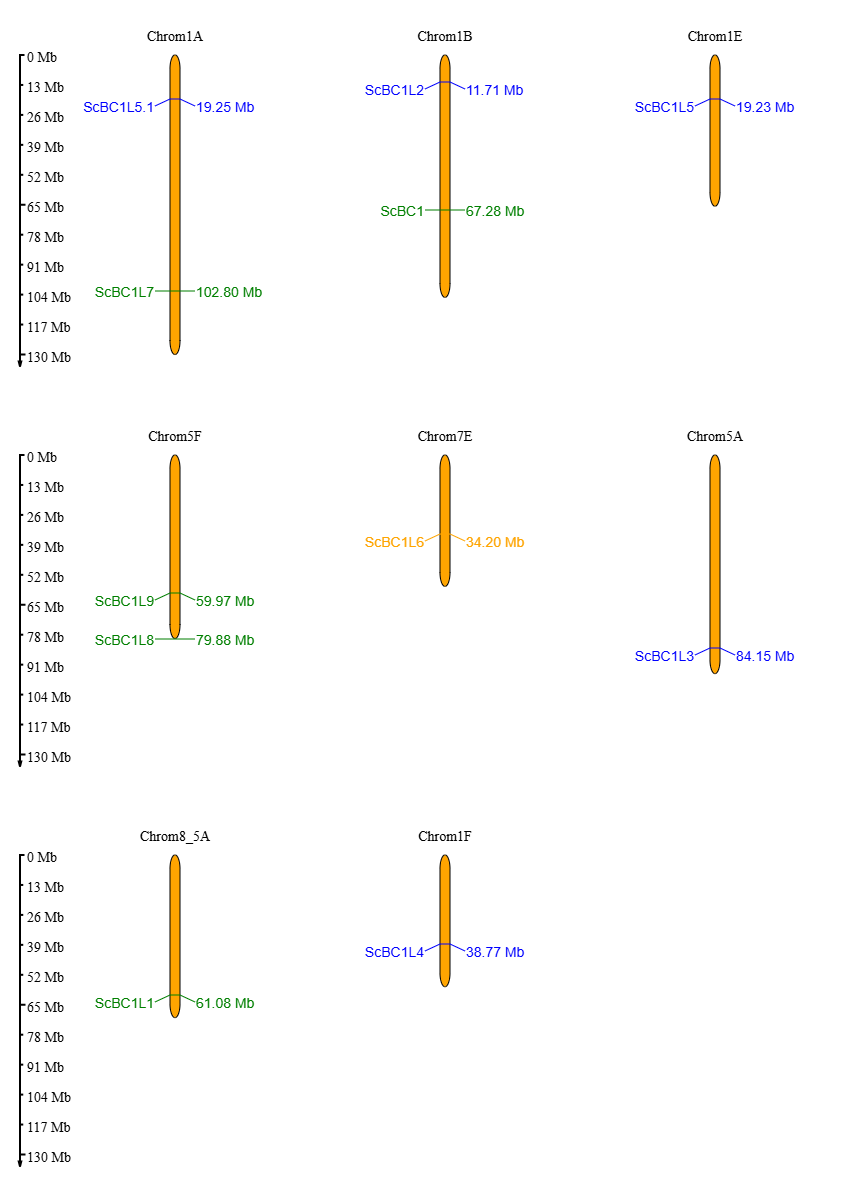


**Supplementary Fig. S3**

**Fig. S3:** Heatmap of the five *COBRA-like* genes (*ScBC1*, *ScBC1L7*, *ScBC1L1*, *ScBC1L3*, and *ScBC1L5.1*) expression in sugarcane SUCEST libraries dataset. Each library corresponds to a distinct tissue: RT1 = Roots or tissues from which roots emerge; LB1 = Apical meristem of young plants and lateral shoots of adult plants; LR1 = Leaf roll of adult plants; SD1 = Developing seeds; AD1 = Seedling tissues cultivated in vitro infected with *Gluconacetobacter diazotroficans* and *Herbaspirillum diazotroficans*; FL4 = Floral tissues; RZ1, RZ3 = Root to shoot zone of young plants grown in a greenhouse; LV = Etiolated leaves of seedlings cultivated in vitro; AM2 = Apical meristem of young plants and lateral shoots of adult plants; SB1 = Stem bark of plants; ST3 = Fourth internodes of adult plants during intense sucrose synthesis and accumulation.


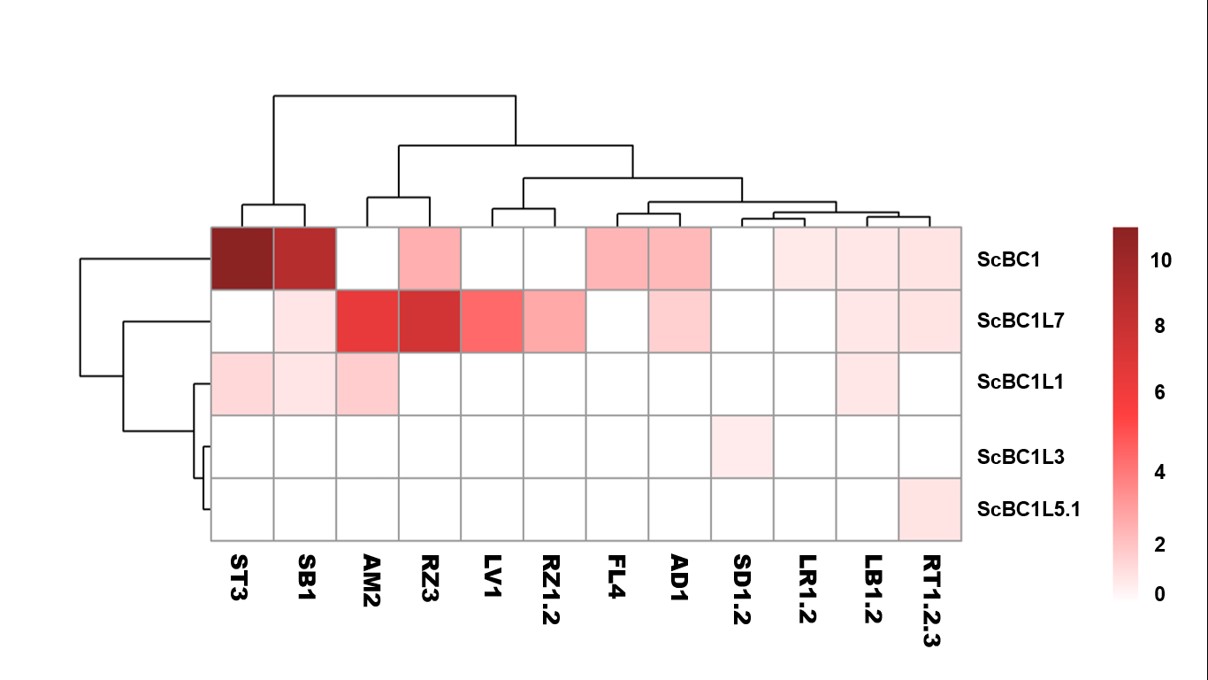


**Supplementary Fig. S4**

**Figure S4:** Heterologous expression in *N. benthamiana* leaves agroinfiltrated with FoMV-VOX-ScBC1L2, FoMV-VOX-ScBC1, pFoMV-DC, and FoMV-VOX-GFP. (a) Bright-field microscopy (BF) images captured at 50 ms exposure and 20× magnification. (b) EYFP filter microscopy images (137.9 ms exposure, 40× magnification) showing GFP expression. (c) Immunoblot assay detecting GFP expression in *N. benthamiana* leaves agroinfiltrated with FoMV-VOX-GFP. The upper panel shows immunoblots probed with an anti-GFP antibody, while the lower panel presents the corresponding protein loading control.


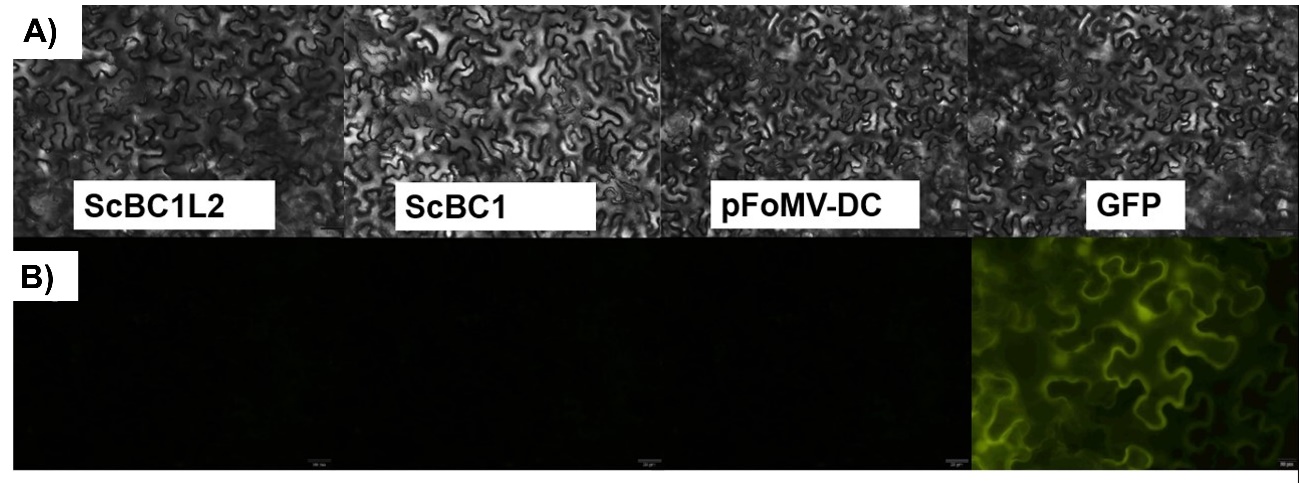


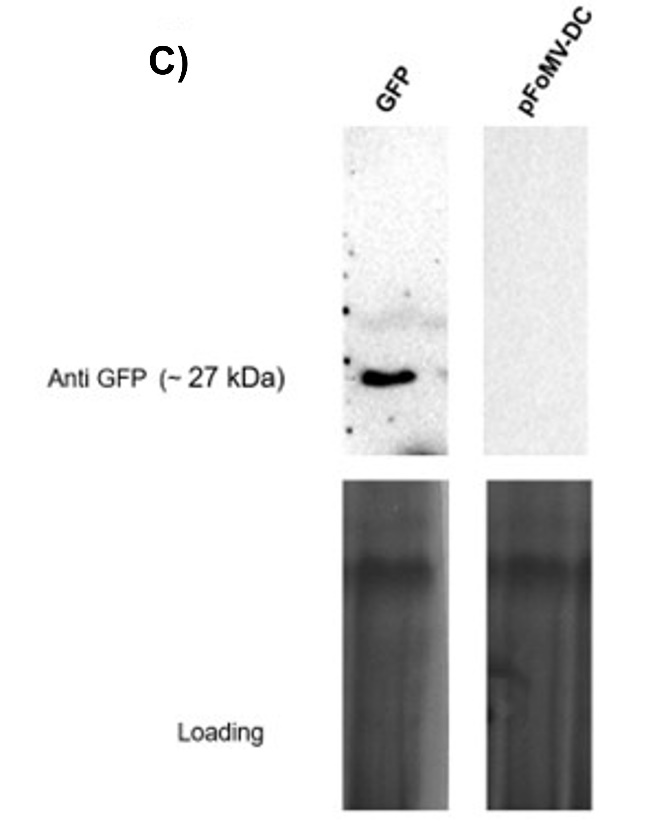


**Supplementary Fig. S5**

**Fig. S5:** Polysaccharide composition of *N. benthamiana* leaves agroinfiltrated with the constructs FoMV-VOX-ScBC1, FoMV-VOX-ScBC1L2, FoMV-VOX-GFP, pFoMV-DC, and wild-type plants, including arabinose (Ara), rhamnose (Rha), xylose (Xyl), mannose (Man), galactose (Gal), and glucose (Glc). (a) Total monosaccharides released after complete acid-hydrolysis of cell wall polysaccharides. (b) Matrix polysaccharides (pectic substances and hemicellulose) released after mild acid-hydrolysis of cell wall polysaccharides. (c) Glucose from crystalline cellulose in the cell wall calculated by difference of total glucose and matrix glucose. Average and SD values are reported. Statistically significant differences between samples were analyzed using One-way ANOVA followed by Tukey’s test (*p* < 0.05).


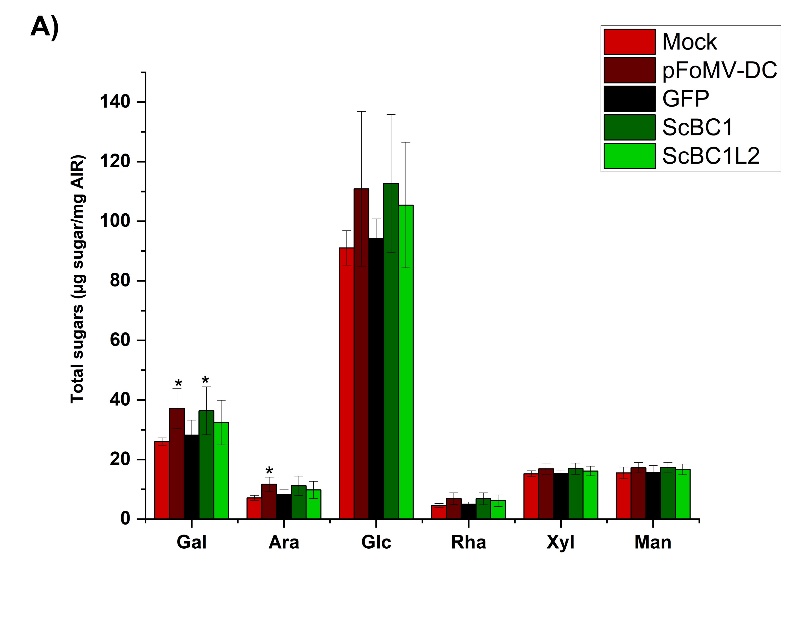


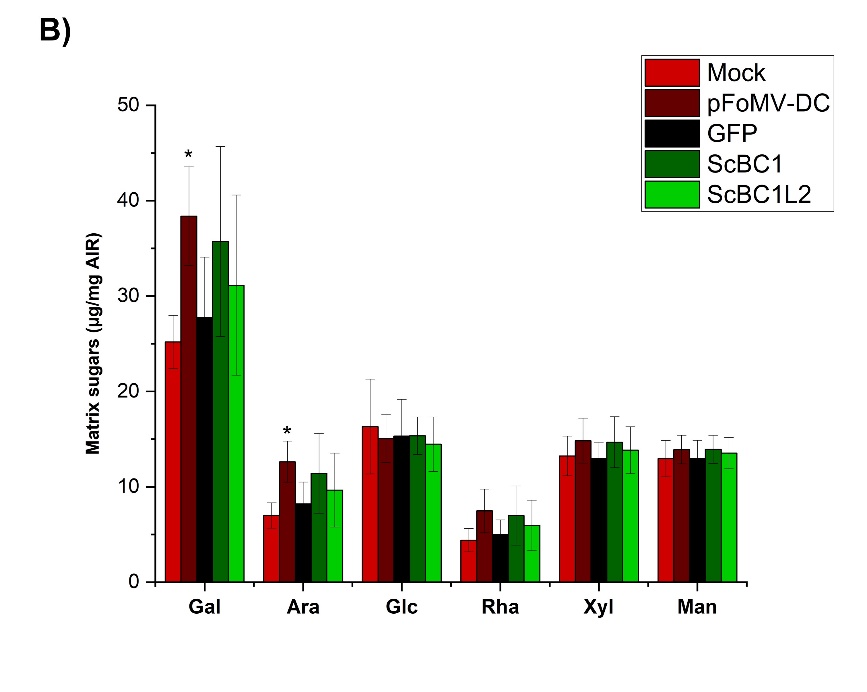


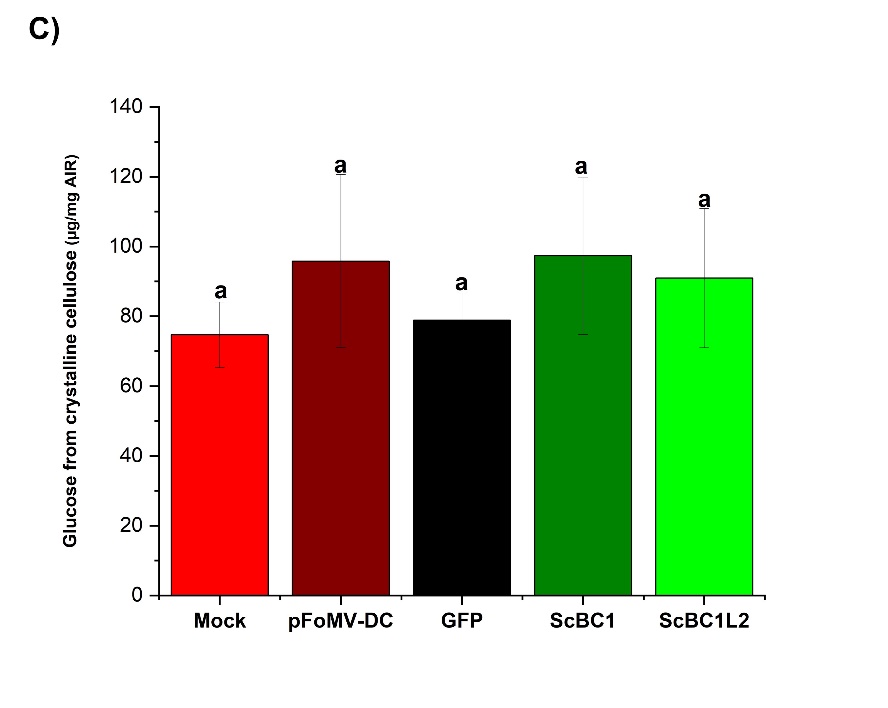


**Supplementary Fig. S6**

**Fig. S6:** RT-PCR amplification of target regions from FoMV-VIGS-ScBC1L2 (573 bp) and FoMV-VIGS-ScBC1 (574 bp) constructs expressed in *N. benthamiana* leaves following agroinfiltration. *ACT09* gene was used as a constitutive internal control.


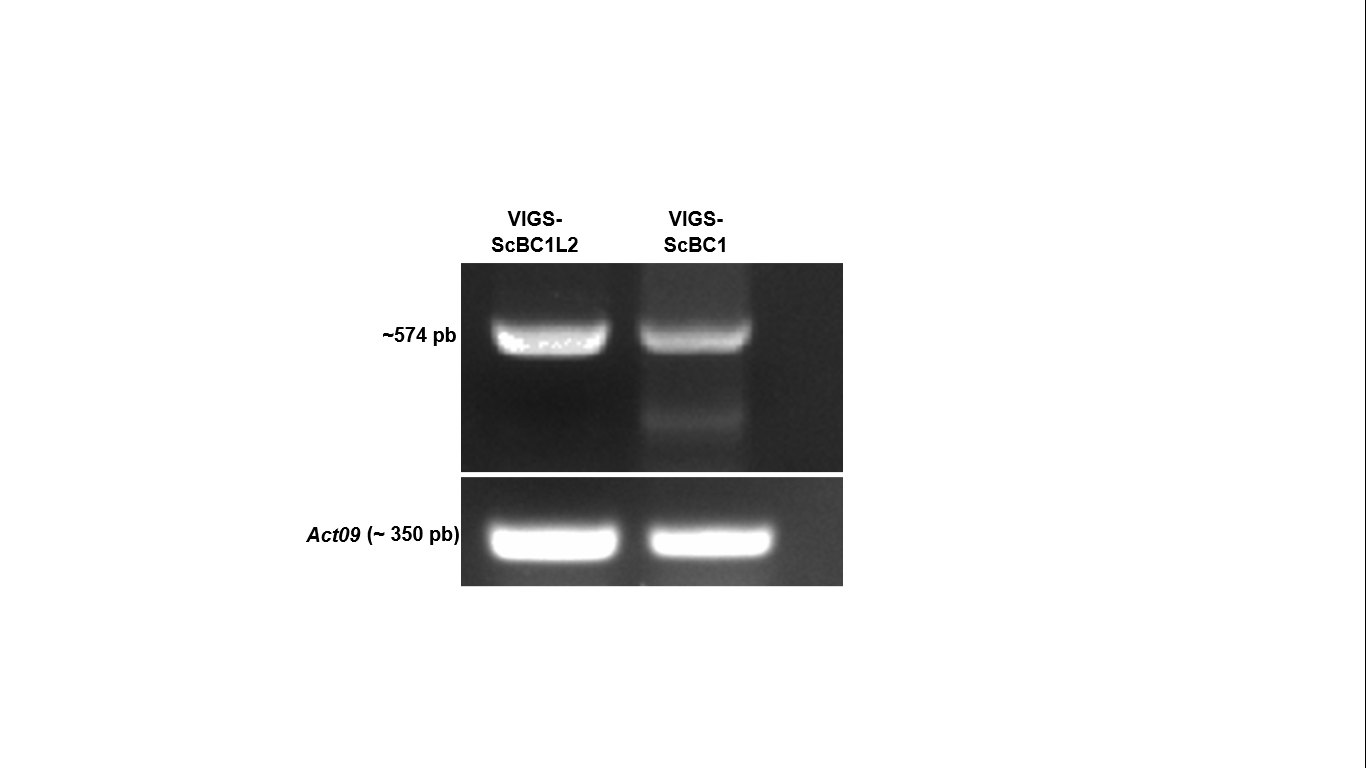


**Supplementary Fig. S7**

**Fig. S7:** Cell wall composition and morphological traits of *ZmBK2* and *ZmBK2L3* VIGs-silenced maize plants. (a) Total glucose (µg/mg alcohol-insoluble residue [AIR]) in leaf cell walls; (b) Glucose from crystalline cellulose (µg/mg AIR); (c) Morphological parameters, including plant height, total leaf area, and leaf dry biomass; (d) Representative image showing the growth of a silenced *ZmBK2L3* plant (*ScBC1L2-1*) and *ZmBK2* (*ScBC1-5*) compared to the Mock control plant. pFoMV-DC: empty vector control; Mock: non-inoculated control. Letters and asterisks indicate statistically significant differences among samples based on one-way ANOVA followed by Tukey’s test (p < 0.05).


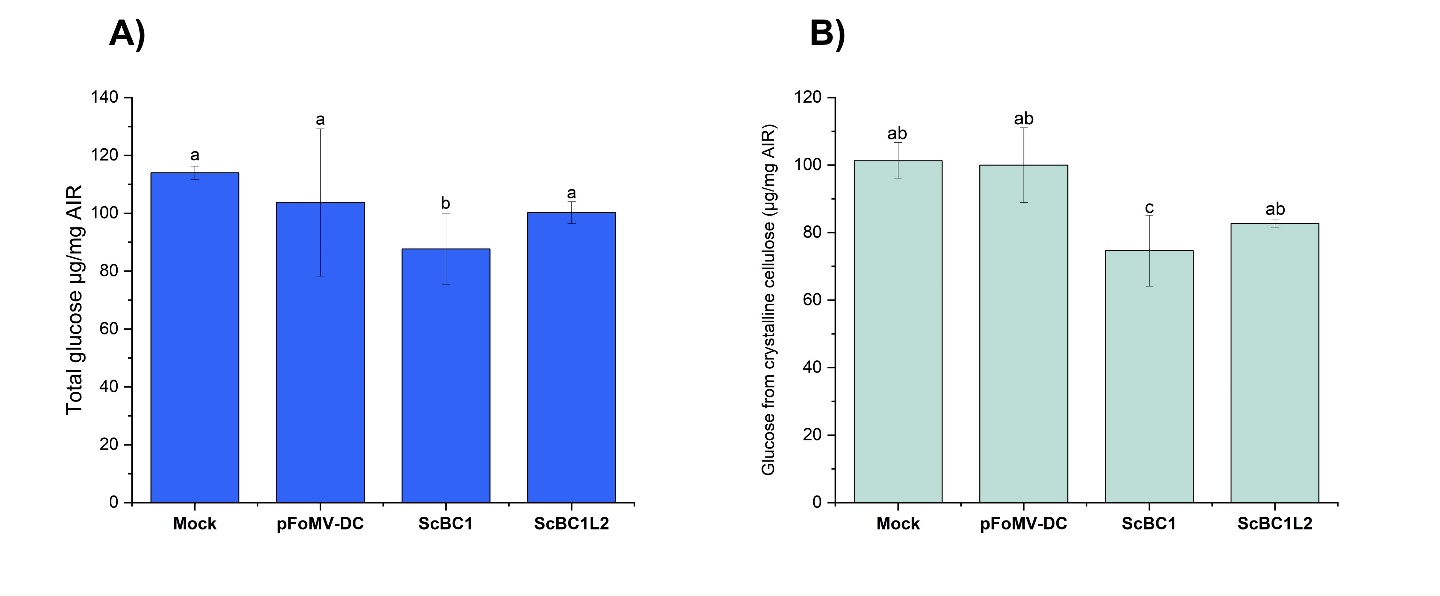


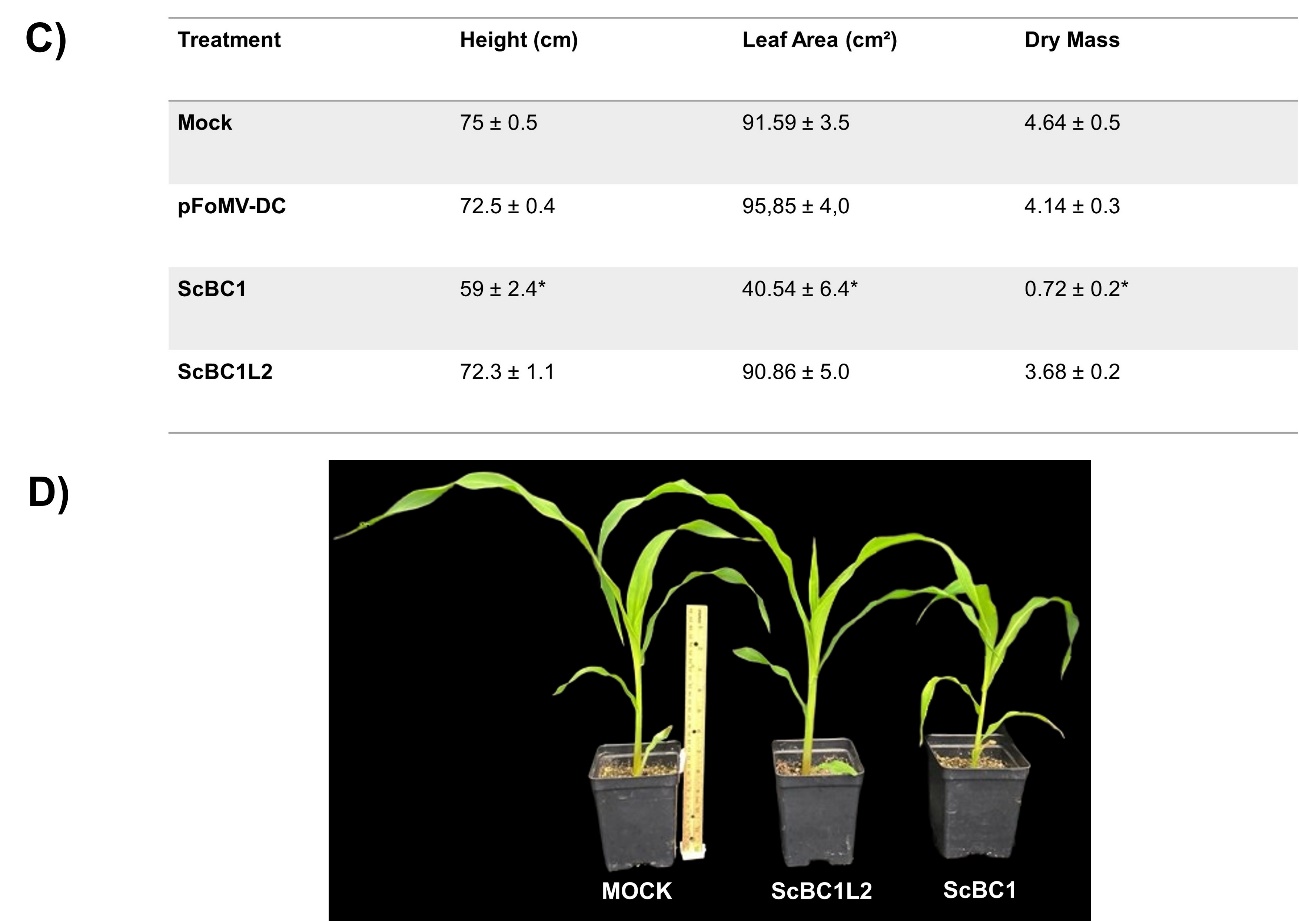


**Supplementary Fig. S8**

**Fig. S8:** Relative expression levels of cellulose synthase genes in *ZmBK2* VIGS-silenced and control maize plants. Transcript levels of secondary cell wall cellulose synthase genes (*ZmCesA1*0*, ZmCesA11*, and *ZmCesA12*) in silenced individuals (ScBC1-2, ScBC1-5, and ScBC1-6) compared to control plants*.* pFoMV-DC: empty vector control; Mock: non-inoculated control. Gene expression was normalized to the reference gene and is presented relative to the average of control samples. Error bars represent standard error. Letters denote statistically significant differences compared to controls (p < 0.05, Student’s t-test or ANOVA as appropriate).


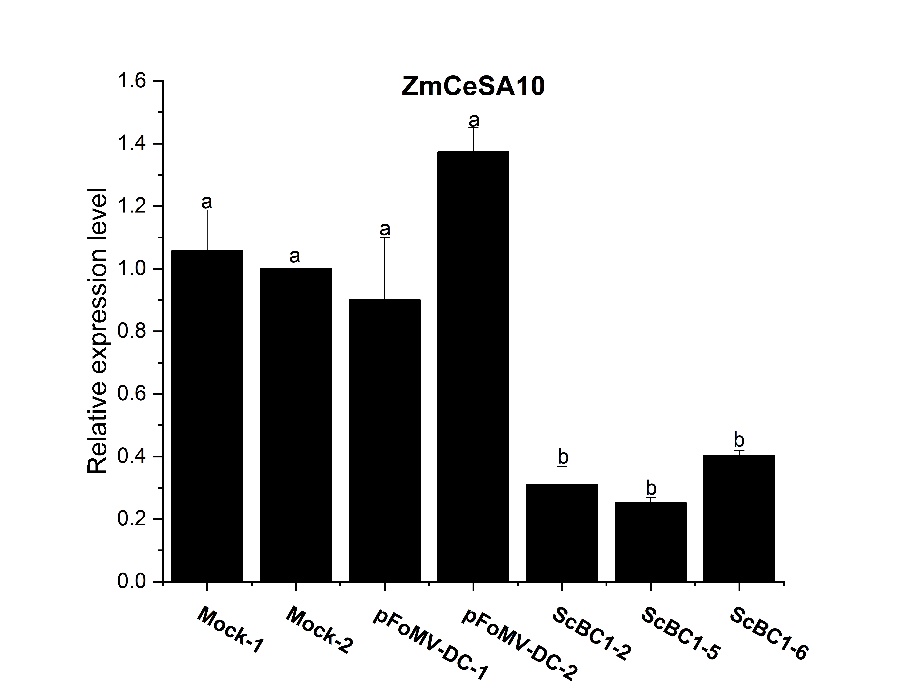


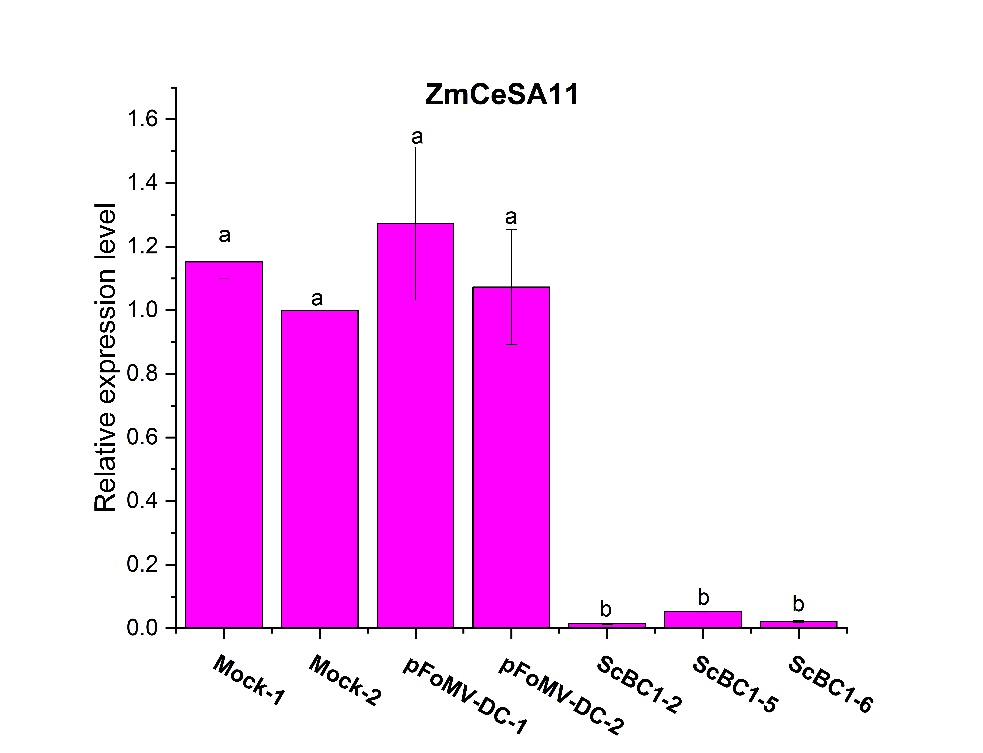


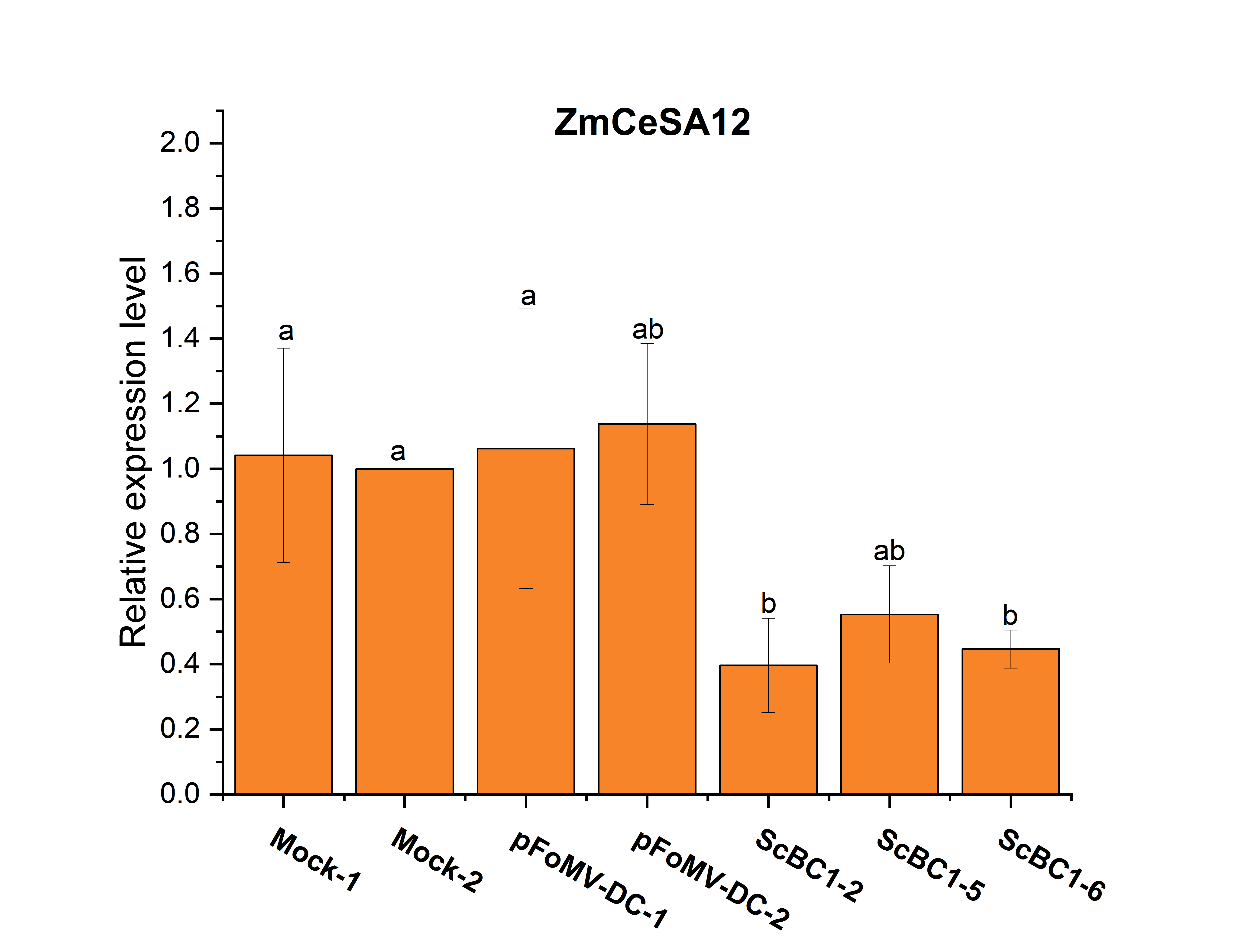

Supplement: Supplementary file 1 — Supplementary Material 1. [file 12870_2025_7910_MOESM1_ESM.docx]
